# Transcription factors ZmNF-YA1 and ZmNF-YB16 regulate plant growth and drought tolerance in maize

**DOI:** 10.1093/plphys/kiac340

**Published:** 2022-07-21

**Authors:** Yaling Yang, Baomei Wang, Jiemin Wang, Chunmei He, Dengfeng Zhang, Peng Li, Juren Zhang, Zhaoxia Li

**Affiliations:** Key Laboratory of Plant Development and Environment Adaptation Biology, Ministry of Education, School of Life Sciences, Shandong University, Qingdao 266237, China; Key Laboratory of Plant Development and Environment Adaptation Biology, Ministry of Education, School of Life Sciences, Shandong University, Qingdao 266237, China; Key Laboratory of Plant Development and Environment Adaptation Biology, Ministry of Education, School of Life Sciences, Shandong University, Qingdao 266237, China; Maize Research Institute, Shandong Academy of Agricultural Sciences, Jinan 250100, Shandong, China; Institute of Crop Sciences, Chinese Academy of Agricultural Sciences, Beijing, 100081, China; Key Laboratory of Plant Development and Environment Adaptation Biology, Ministry of Education, School of Life Sciences, Shandong University, Qingdao 266237, China; Key Laboratory of Plant Development and Environment Adaptation Biology, Ministry of Education, School of Life Sciences, Shandong University, Qingdao 266237, China; Key Laboratory of Plant Development and Environment Adaptation Biology, Ministry of Education, School of Life Sciences, Shandong University, Qingdao 266237, China

## Abstract

The identification of drought stress regulatory genes is crucial for the genetic improvement of maize (*Zea mays* L.) yield. Nuclear factors Y (NF-Ys) are important transcription factors, but their roles in the drought stress tolerance of plants and underlying molecular mechanisms are largely unknown. In this work, we used yeast two-hybrid screening to identify potential interactors of ZmNF-YB16 and confirmed the interaction between ZmNF-YA1 and ZmNF-YB16-YC17 and between ZmNF-YA7 and ZmNF-YB16-YC17. ZmNF-YB16 interacted with ZmNF-YC17 *via* its histone fold domain to form a heterodimer in the cytoplasm and then entered the nucleus to form a heterotrimer with ZmNF-YA1 or ZmNF-YA7 under osmotic stress. Overexpression of *ZmNF-YA1* improved drought and salt stress tolerance and root development of maize, whereas *zmnf-ya1* mutants exhibited drought and salt stress sensitivity. ZmNF-YA1-mediated transcriptional regulation, especially in JA signaling, histone modification, and chromatin remodeling, could underlie the altered stress tolerance of *zmnf-ya1* mutant plants. ZmNF-YA1 bound to promoter CCAAT motifs and directly regulated the expression of multiple genes that play important roles in stress responses and plant development. Comparison of ZmNF-YB16- and ZmNF-YA1-regulated genes showed that ZmNF-YA1 and ZmNF-YB16 have similar biological functions in stress responses but varied functions in other biological processes. Taken together, ZmNF-YA1 is a positive regulator of plant drought and salt stress responses and is involved in the root development of maize, and ZmNF-Y complexes with different subunits may have discrepant functions.

## Teaser

Transcription factors ZmNF-YA1 and ZmNF-YB16 play positive roles in plant growth and drought tolerance in maize via directly regulating expression of multiple genes.

## Introduction

Abiotic stresses, for example, drought, salinity, heat/chilling stress, and nutrient deficiency, seriously affect the growth and development of plants. After perceiving stress signals, plants activate a series of signal transduction pathways and respond to stresses. During these processes, transcription factors (TFs) regulate a number of stress-responsive genes belonging to different families.

Nuclear factor Y (NF-Y), also called heme activate protein (HAP) or CCAAT box combination factor (CBF), is a heterotrimeric TF consisting of three evolutionarily distinct NF-YA (CBF-B or HAP2), NF-YB (CBF-A or HAP3), and NF-YC (CBF-C or HAP5) subunits, which are conserved across eukaryotic lineages (Sinha et al., 1995). In yeast, assembly of the NF-Y heterotrimeric complex occurs in a strictly ordered manner. NF-YB and NF-YC, the histone-like subunits, form a heterodimer in the cytoplasm, which subsequently translocates into the nucleus, where NF-YB/-YC interacts with NF-YA to form an NF-Y heterotrimer (Romier et al., 2003; Frontini et al., 2004). The trimer recognizes and binds with high affinity and sequence specificity to the cis-acting element CCAAT motif frequently observed in eukaryotic promoters (Xing et al., 1993). The NF-Y TF can either transcriptionally activate or repress downstream genes, and interactions with other regulatory factors can also regulate its activity. Each NF-Y family has only one member in yeast and animals. In plants, however, each type of NF-Y subunit has multiple genes. Genome-wide analysis of NF-Ys in Arabidopsis (*Arabidopsis thaliana*) revealed 10, 13, and 13 genes encoding NF-YA, -YB and -YC subunits, respectively (Siefers et al., 2009). In maize (*Zea mays* L.), 14 NF-YA, 18 NF-YB, and 18 NF-YC genes were identified (Zhang et al., 2016).

In plants, NF-Y genes present multifarious expression patterns during different developmental stages or in response to environmental stresses, suggesting that combining different subunits can assemble NF-Y complexes with diversified functions. The members of the NF-Y gene family play varied roles in plant developmental processes and stress responses, such as embryogenesis, seed development and photoperiod-dependent flowering. NF-Ys are also involved in plant growth control. The miR169 and their targets *AtNF-YA2* and *AtNF-YA10* are expressed in root vascular tissues and act to control apical root meristem and lateral root initiation (Sorin et al., 2014). Overexpression (OE) of *AtNF-YB2* (HAP3b) promotes primary root elongation by accelerating cell division and/or elongation (Ballif et al., 2011); OE of *TaNF-YA-B1* in wheat (*Triticum aestivum*) enhances root development by increasing lateral root growth and increases both nitrogen and phosphorus uptake leading to increased grain yield in wheat (Qu et al., 2015). OE of *OsHAP3E* results in a dwarf phenotype with erect leaves and aberrant panicles, whereas repression of *OsHAP3E* leads to lethality, indicating pleiotropic roles in vegetative and reproductive development in rice (*Oryza sativa*; Ito et al., 2011; Zhang and Xue, 2013). Knockdown of *LjNF-YA1* inhibits root nodule formation, and ectopic expression of *LjNF-YA1* and *LjNF-YB1* causes abnormal cell division in lateral root primordia, indicating that the NF-Y subunits are associated with root nodule organogenesis and lateral root formation in *Lotus japonicas* (Soyano et al., 2013). Arabidopsis NF-YB2/NF-YC3 and CONSTANS, a master flowering regulator, form a trimer to efficiently bind the core element of the *FLOWERING LOCUS T* promoter to regulate the timing of flowering (Gnesutta et al., 2017). In rice, NF-YC12 and bHLH144 maintain NF-YB1 stability from degradation mediated by ubiquitin/26S proteasome, whereas NF-YB1 directly binds to the “G-box” domain of the *Waxy* promoter and activates *Waxy* transcription, hence regulating grain quality (Bello et al., 2019). NF-Ys have also been identified as regulators of abiotic stress tolerance in different plant species. Transgenic Arabidopsis overexpressing *AtNF-YB1* and transgenic maize overexpressing the putative ortholog gene *ZmNF-YB2* present elevated drought tolerance (Nelson et al., 2007). The expression of *AtNF-YA5*, which miR169 can post-transcriptionally regulate, is induced by drought stress in an abscisic acid (ABA)-dependent manner (Li et al., 2008b). OE of *AtNF-YA5* improves drought tolerance by regulating the expression of stress-responsive genes (Li et al., 2008b). OE *AtNF-YA2, -YA3, -YA7*, or *-YA10* lines show higher tolerance to abiotic stresses, including hypoxia, drought, heat, and cold stresses (Leyva-González et al., 2012). Transgenic Arabidopsis overexpressing *GmNF-YA3* from soybean shows enhanced ABA and salt sensitivity and improved drought tolerance (Ni et al., 2013). *AtNF-YA1* negatively regulates seed germination and postgermination growth under salt stress (Li et al., 2013). OE of *OsNF-YA2* (*OsHAP2E*) in rice showed resistance to biotic stress, tolerance to salinity and drought, increased photosynthesis and tiller number (Alam et al., 2015). Two NF-Y genes from foxtail millet (*Setaria italica*), *SiNF-YA1* and *SiNF-YB8*, improve stress tolerance in Tobacco W38 (*Nicotiana tabacum*) by activating stress-related genes and improving physiological traits through ABA-dependent and reactive oxygen species signaling pathways (Feng et al., 2015). Screening of activation-tagged indica rice line responses to salt stress identified a salt-tolerant line DS-16 in which the expression of a gene encoding the NF-YC subunit (OsNF-YC13) was induced (Manimaran et al., 2017). In Arabidopsis, the endoplasmic reticulum (ER) stress response of the TF component of bZIP28 interacts with a NF-Y heterotrimeric protein complex consisting of NF-YA4, NF-YB3, and NF-YC2 (Liu and Howell, 2010). NF-YA2, NF-YB3, and NF-YC10 can synergistically activate a promoter of the heat stress-inducible gene with DREB2A in Arabidopsis protoplasts (Sato et al., 2014).

In maize, 50 ZmNF-Y genes were identified with tissue-specific expression patterns in different development stages and responses to various abiotic and biotic stresses (Zhang et al., 2016). Luan et al. reported that zma-miR169 genes and their target *ZmNF-YA* genes respond to drought, ABA and salt stresses in maize leaves and roots (Luan et al., 2014; Luan et al., 2015). ZmNF-YA3 also interacts with CONSTANS-like (CO-like) and flowering promoting factor1 (FPF1), and the complex can regulate flowering time (Su et al., 2018). ZmNF-YC2 in maize under long-day conditions is a positive regulator of flowering time by promoting the expression of ZmNF-YA3, the latter negatively regulates the transcription of ZmAP2 that suppresses the expression of the MADS-box gene ZMM4 to delay flowering time (Su et al., 2021). These studies indicate that some members of ZmNF-Y may play important roles in plant development and the response and adaptation to stresses; however, the biological roles of ZmNF-Y genes in maize are still very limited. NF-YB plays a vital role in the specific binding of the NF-YA, NF-YB, and NF-YC trimer to promoters (Zemzoumi et al., 1999). NF-YB gene family has been expanded in plants (Siefers et al., 2009), suggesting that their function diversity was more complex than previously thought. Our previous work showed that OE of *ZmNF-YB16* improves drought tolerance and yield by enhancing photosynthesis and the antioxidant capacity of maize plants (Wang et al., 2018). Expression analysis showed that ZmNF-YB16 regulates the expression of genes encoding antioxidant enzymes, the antioxidant synthase, and molecular chaperones associated with the ER stress response and improves the protection of photosynthesis system II. Being an important transcriptional regulation internode that contributes to stress response and confers to crop yield, understanding the functional differences between *ZmNF-YB16* and other subfamily members would be beneficial. It would also be important to elucidate how the OE of *ZmNF-YB16* leads to the improvement of plant drought tolerance and yield and which partners with *ZmNF-YB16* form a complex to function as a regulator. To address these questions, we sought the other members of a heterotrimer with ZmNF-YB16 and found that ZmNF-YB16 interacts with ZmNF-YC17 *via* its histone fold domain and then forms a heterotrimer with ZmNF-YA1 when subjected to osmotic stress. The *ZmNF-YA1* OE and mutant lines were used to explore the effects and mechanism of ZmNF-YA1 on maize growth and abiotic stress tolerance in this paper.

## Results

### ZmNF-YB16 interacts with ZmNF-YC17

**Figure 1:**
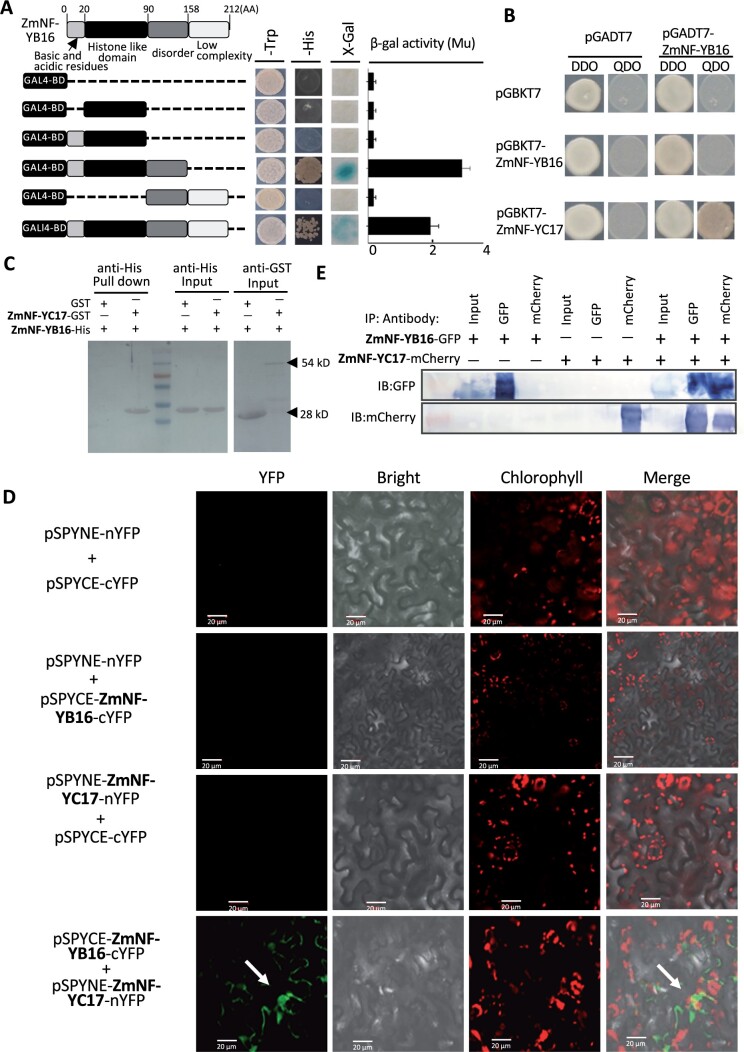
ZmNF-YB16 interact with ZmNF-YC17 in vitro and in vivo. A, Transcriptional activation analysis of ZmNF-YB16. Numbers at the top indicate the position of AAs. The N-terminal 21–90 AA represents the histone-like domain. Different lengths of proteins were cloned and fused to the GAL4 DNA binding (BD) vector. Transformants were screened on SD/-Leu and SD/-His, and β-galactosidase (β-gal) activities were validated by X-Gal staining and quantified by the ONPG assay. B, Growth of yeast cells harboring ZmNF-YB16 and ZmNF-YC17 fused to the GAL4 BD domain (pGBKT7-BD) and GAL4 activity domain (pGADT7-AD). SD/-Leu/-Trp (DDO) is the nonselective medium, and SD/-Leu/-Trp/-Ade/-His (QDO) is the selective medium. C, In vitro pull-down assay of ZmNF-YC17-GST protein and ZmNF-YB16-His protein. The fractions pulled down were detected with an His antibody. D, Verification of the in vivo interaction between ZmNF-YB16 and ZmNF-YC17 by the BiFC system in *N. benthamiana*. Expression of ZmNF-YB16-cYFP and ZmNF-YC17-nYFP was observed in leaves. The YFP fluorescence signal was detected in the group co-transformed of ZmNF-YB16-cYFP and ZmNF-YC17-nYFP (lower row), but not in the control groups (Rows 1–3). *bar* = 20 µm. E, Co-IP assay to test the direct interaction between ZmNF-YB16 and ZmNF-YC17. IP: antibody, antibody used for immunoprecipitation, IB: antibody, antibody used for western blot.

To identify the proteins that interacted with ZmNF-YB16, we performed a yeast two-hybrid screen. First, we tested the transcriptional activity of ZmNF-YB16. As shown in Figure 1A, ZmNF-YB16 contained a histone-like domain (21–90 amino acid [AA]). The N-terminal 90 AA and C-terminal 122 AA had no transcriptional activity. However, the full-length ZmNF-YB16 and N-terminal 158 AA showed transcriptional activity. The transcriptional activity of the N-terminal 158 AA was stronger than that of the full-length ZmNF-YB16. The activity of full-length ZmNF-YB16 was 1.96 Mu, and that of the N-terminal 158 AA was 2.78 Mu measured by β-gal activity (Figure 1A).

The full-length ZmNF-YB16 was fused to the yeast GAL4 DNA binding domain and used as bait. Approximately 9.5 × 10^7^ cfu/mL yeast transformants were screened, and after verification of growth on a synthetic medium lacking Leu/His/Trp/Ade (QDO), six clones containing the ZmNF-YC17 (GRMZM2G311316) cDNA were identified (Figure 1B). We further tested the direct interaction between ZmNF-YB16 and ZmNF-YC17 using an in vitro pull-down assay. As a result, ZmNF-YB16-His was detected in the ZmNF-YC17-GST fraction but not in the GST alone fraction (Figure 1C and Supplemental Figure S1). These data indicated that ZmNF-YB16 interacted directly with ZmNF-YC17, and the presence of the histone-like domain together with the disorder domain are required for transcriptional activity in this assay.

We then analyzed the interaction between ZmNF-YC17 and ZmNF-YB16 in *Nicotiana benthamiana* by using the bimolecular fluorescence complementation (BiFC) system and the co-immunoprecipitation (Co-IP) assay (Figure 1, D and E). As a result, the fluorescence signal was detected in the group co-transformed of ZmNF-YB16-cYFP and ZmNF-YC17-nYFP, but not in the control groups (Figure 1D). These data indicated that ZmNF-YB16 interacted with ZmNF-YC17 in vivo. The direct interaction between ZmNF-YB16 and ZmNF-YC17 using Co-IP assay was detected (Figure 1E).

**Figure 2:**
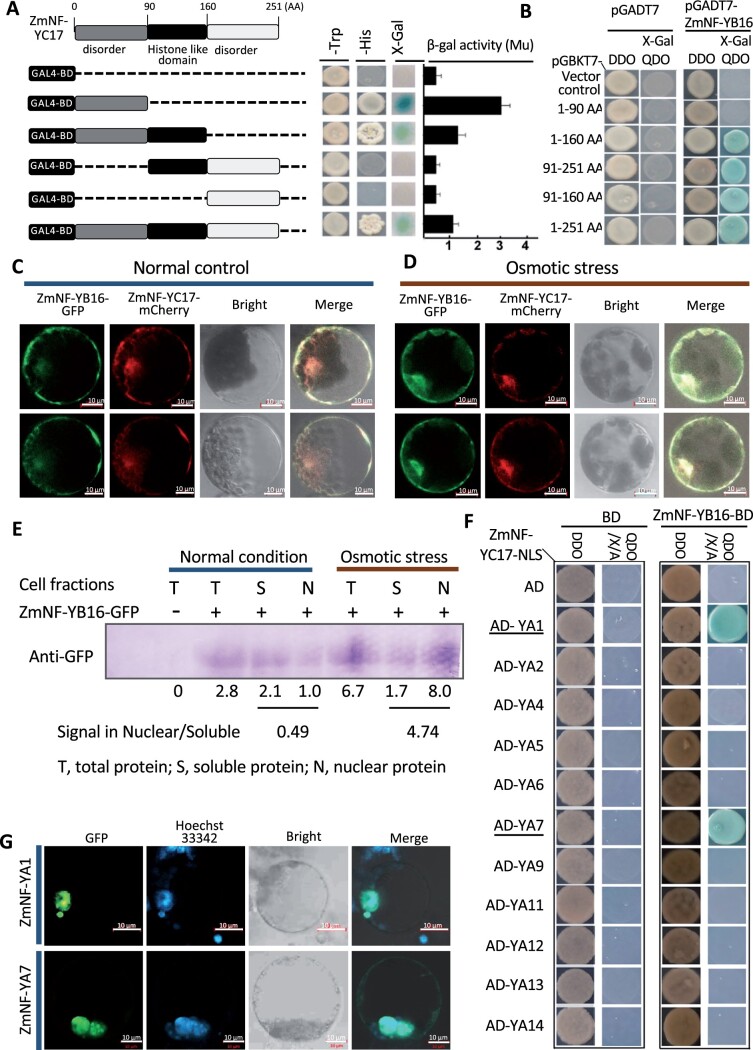
ZmNF-YB16/ZmNF-YC17/ZmNF-YA1/7 form heterotrimers in maize. A, Transcriptional activation analysis of ZmNF-YC17. Numbers at the top indicate the position of AAs. The N-terminal 91–160 AA represents the histone-like domain region. The different lengths of proteins were cloned and fused to the GAL4 BD vector. Transformants screen, β-gal activities, X-Gal staining, and ONPG quantification were performed as that in Figure 1A. B, Growth of yeast cells harboring various truncated forms of ZmNF-YC17 fused to the GAL4 BD vector. ZmNF-YB16 was fused to the GAL4 AD vector. Transformants screen, X-Gal staining were performed as that in Figure 1B. C, Subcellular localization analysis of ZmNF-YB16 and ZmNF-YC17 under normal condition. *bar* = 10 µm. D, Subcellular colocalization analysis of ZmNF-YB16 and ZmNF-YC17 under osmotic stress conditions. ZmNF-YB16 was fused to the pUC18-P35S-*GFP* vector, and ZmNF-YC17 was fused to the pUC18-P35S-*mCherrry* vector. *bar* = 10 µm. E, Confirmation of subcellular location changes by cytosol and nuclear fraction and western-blot. Relative expression levels were quantified using Image J. F, Yeast three-hybrid assay to identify candidate trimers containing ZmNF-YB16 and ZmNF-YC17. The growth of yeast cells harboring NF-YA family proteins fused to the GAL4 AD, ZmNF-YB16 fused to the GAL4 BD, and ZmNF-YC17 fused to a NLS on selective medium. Transformants screen, X-Gal staining were performed as that in Figure 1B. G, Subcellular localization of ZmNF-YA1 and ZmNF-YA7 under normal conditions. ZmNF-YA1 and ZmNF-YA7 were fused to the pUC18-P35S-*GFP* vector, respectively. *bar* = 10 µm.

ZmNF-YC17 fragments were ligated into the GAL4-BD vector according to the function domain to identify the interacting regions between them. Figure 2A shows that the histone-like domain of ZmNF-YC17 had no transcriptional activity, in contrast to the N-terminal 90 AA. The transcriptional activity of the full-length ZmNF-YC17 was much weaker than that of the N-terminal 90 AA (Figure 2A). The yeast-hybrid assay showed that ZmNF-YC17 interacted with ZmNF-YB16 via their histone-like domains (Figure 2B).

### The location of the ZmNF-YB16 and ZmNF-YC17 is altered under different conditions

The subcellular location assay revealed the expression of ZmNF-YB16 and ZmNF-YC17 in the cytoplasm in W1 solution for protoplast incubation (Figure 2C). Because ZmNF-YB16 interacted with ZmNF-YC17 in vitro and in vivo, a colocalization assay was necessary. When the protoplasts transformed with ZmNF-YB16-GFP and ZmNF-YC17-mCherry were incubated in the W1 solution supplemented with 0.6 mol mannitol (osmotic stress), the fluorescent signal was detected in the cytoplasm to the nucleus, and ZmNF-YB16 showed almost complete colocation with ZmNF-YC17 (Figure 2D). We further confirmed the subcellular location changes by western-blot with cytosol and nuclear fraction, respectively (Figure 2E). These results implied that ZmNF-YB16 in osmotic stress response depended on the translocation of the ZmNF-YB16 from the cytoplasm to the nucleus. Maize ZmNF-YB16 may maintain the basic characteristics of its mammalian homologs, such as heterodimer formation with NF-YB and NF-YC proteins and subsequent translocation from the cytosol to the nucleus (Frontini et al., 2004; Kahle et al., 2005), although we still lack the knowledge how they were translocated.

### ZmNF-YB16 and ZmNF-YC17 form heterotrimers with ZmNF-YA1/7

Some studies have suggested that NF-YA, NF-YB, NF-YC form a complex to regulate target gene expression (Laloum et al., 2013; PetroNi et al., 2013). To determine which members of the NF-YA family form a trimer with the ZmNF-YB16/YC17 complex, the genes of all members of the ZmNF-YA family were analyzed by constructing the evolutionary tree (Supplemental Figure S2). Fourteen members in the ZmNF-YA family produced 86 kinds of transcripts, with each gene encoding several transcripts except the *ZmNF-YA11* gene. Among them, the genes coding ZmNF-YA1, -YA2, -YA4, -YA5, -YA7, -YA9, -YA11, -YA12, -YA13, and -YA14 were cloned and inserted into the GAD4-AD vector, respectively. *ZmNF-YB16* and *ZmN-YC17* fused to the nuclear localization signal (NLS) were recombined in the GAL4-BD vector. The three-hybrid system in yeast was used to identify ZmNF-YA family members that could interact with the ZmNF-YB16-YC17 complex. ZmNF-YA1 and ZmNF-YA7 could interact with the ZmNF-YB16-YC17 heterodimer, respectively, but other members could not (Figure 2F). The subcellular location assay also showed ZmNF-YA1 and ZmNF-YA7 localization in the nucleus (Figure 2G). The results indicated that the ZmNF-YA1-YB16-YC17 and ZmNF-YA7-YB16-YC17 trimers could be formed in the nucleus, implying that upon osmotic stress, ZmNF-YB16 and ZmNF-YC17 was transferred from the cytoplasm to the nucleus and therein formed a trimer with ZmNF-YA1 or ZmNF-YA7 to exert its biological function. According to the evolutionary tree analysis (Supplemental Figure S2), the genetic distance between some transcripts of ZmNF-YA1 and ZmNF-YA7 was the closest, and thus, they may have the same or similar functions. The core domains of ZmNF-YA1/7 included an NF-YB/C interaction motif in the N-terminus, a motif recognizing and binding to the CCAAT cis-element in the C-terminus, and a middle linker region, which were also conserved in NF-YA family members (Supplemental Figure S3A). According to the multiple sequence alignment, ZmNF-YA1 and ZmNF-YA7 had a highly conserved sequence in the NF-YB/C binding region and DNA binding domain, but the N-terminal sequence and C-terminal sequence varied greatly (Supplemental Figure S3B). *ZmNF-YA7* (GRMZM2G040349, also named *ZmNF-YA3*) has dual functions in the stress response and the photoperiod-dependent flowering of maize (Su et al., 2018). We further investigated the role of ZmNF-YA1-YB16-YC17 in maize.

### Expression patterns of *ZmNF-YA1* and its partner genes

The organ expression patterns of *ZmNF-YA1*, *ZmNF-YB16*, and *ZmNF-YC17* were analyzed according to published works (Winter et al., 2007; Sekhon et al., 2011; Stelpflug et al., 2016) and confirmed by reverse transcription-quantitative PCR (RT-qPCR). As shown in Supplemental Figure S4, *ZmNF-YA1* was ubiquitously expressed in all tissues, but the expression levels varied. *ZmNF-YA1* showed continuously increasing expression models during kernel development and had the highest expression level in the embryo 22 d after pollination (DAP). *ZmNF-YB16* had the highest expression level in leaf tips at the V7 stage, whereas *ZmNF-YC17* had the highest expression level in roots 3 d after sowing (DAS). It is noteworthy that *ZmNF-YC17* maintained high expression levels in leaves from the three DAS stage to the 30 DAP stage. To explore the expression profile of the complex members under stress conditions, we collected leaf samples at different times after osmotic, salt and heat stress treatment (Supplemental Figure S5). *ZmNF-YA1* was induced in all three stress treatments but the trends varied. *ZmNF-YC17* was not substantially induced by osmotic stress, whereas *ZmNF-YB16* and *ZmNF-YA1* were substantially induced in a variable manner. *ZmNF-YB16* had the highest expression levels at 4 h after osmotic stress treatment, whereas *ZmNF-YA1* expression peaked after 24 h after osmotic stress treatment. The expression patterns showed that *ZmNF-YB16* and *ZmNF-YA1* might play important roles in regulating the function of the heterotrimer. OE of *ZmNF-YB16* improved maize plants' drought tolerance and increased the grain yield (Wang et al., 2018). Being the DNA binding subunit of the heterocomplex, ZmNF-YA1 was selected for further analysis using the *zmnf-ya1* mutant and the *ZmNF-YA1* OE lines.

### Plant growth and root development of the *zmnf-ya1* mutant

**Figure 3:**
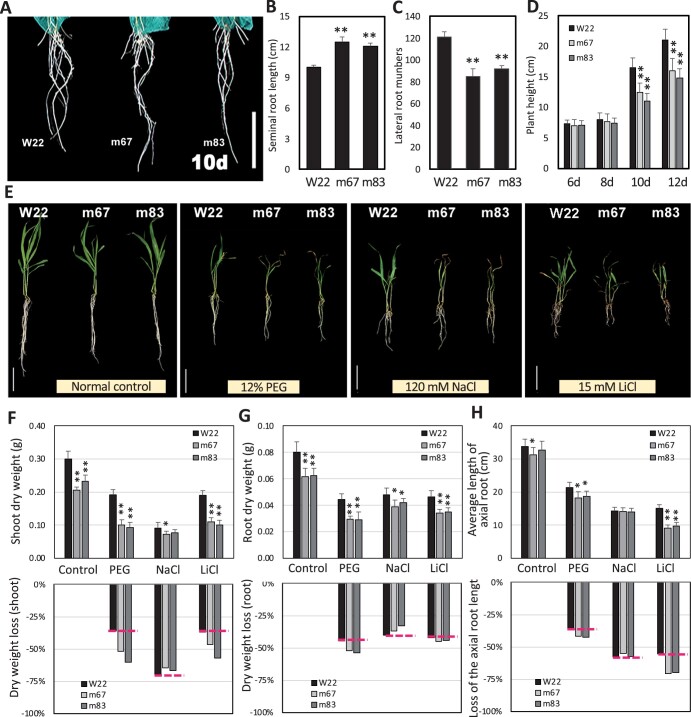
Loss of function of *ZmNF-YA1* suppresses plant growth and root development in maize. A, Root phenotypes of W22 and *zmnf-ya1* mutant plants hydroponically cultured for 10 d. B and C, Total axial root length and lateral root numbers of W22 and *zmnf-ya1* mutant plants hydroponically cultured for 10 d. D, Plant height of WT and mutant plants hydroponically cultured for 5, 8, 10, and 12 d. E, Eleven-day-old hydroponically cultured maize plants under normal conditions (Normal Control) and treated with 12% PEG_6000_, 120 mM NaCl, or 15 mM LiCl for 10 d. F–H, Shoot dry weight, root dry weight and axial root length of WT and *ZmNF-YA1* mutants under different conditions and the loss led by stresses. The images of panel A and E were digitally extracted for comparison. All values are means ± SD of three independent experiments (each with five seedlings per line). *bar* = 5 cm. The asterisks indicate a significant difference at *0.01 < P ≤ 0.05, **P ≤ 0.01 by a *t* test.

Two *zmnf-ya1* homozygous mutant lines, m67 and m83, which had no *ZmNF-YA1* transcripts, were used as material **(**Supplemental Figures S6 and S7). Under normal conditions, the growth of *zmnf-ya1* mutants was weaker than inbred line W22 (Figure 3, A–D). The seminal root length increased, but the number of lateral roots and plant height was reduced in the mutants compared with W22 (Figure 3, B–D). When the phenotypes of the WT and *zmnf-ya1* mutants were characterized under different abiotic stresses, 11-d-old (11 d) hydroponically cultured maize plants were treated with 12% PEG (osmotic stress), 120 mM NaCl (salt stress), or 15 mM LiCl (ion toxicity) for 10 d, respectively. The *zmnf-ya1* mutants grew worse than W22 under these stresses (Figure 3E). For example, PEG treatment decreased the shoot biomass by 42% in W22 compared with normal conditions and 50%–60% in *zmnf-ya1* mutants (Figure 3F). The axial root growth of all seedlings was significantly inhibited by PEG and NaCl treatment, and z*mnf-ya1* mutants exhibited much greater inhibition than W22, especially following treatment with 15 mM LiCl (Figure 3, F–H). Compared with ion toxicity, the z*mnf-ya1* mutants were more sensitive to osmotic stress based on biomass loss (Figure 3, F and G). The growth rates of *zmnf-ya1* mutants were lower than W22, and the plant height, leaf length, leaf width, and leaf area of *zmnf-ya1* mutants at the V5 stage were significantly decreased compared with W22 (Supplemental Figures S6 and S7). These findings demonstrated that *ZmNF-YA1* played important roles in both plant growth and abiotic stress tolerance.

### OE of *ZmNF-YA1* improves plant growth and tolerance to abiotic stresses

*ZmNF-YA1* OE plants were produced by *Agrobacterium tumefaciens*-mediated maize shoot-tip transformation. According to PCR and Southern blotting analyses, transgenic fragments were stably inserted into the maize genome (Supplemental Figure S7, D–F). The expression assay showed *ZmNF-YA1* transcript levels were significantly upregulated by osmotic stress in the OE lines since the RD29A promoter was used to drive the expression of *ZmNF-YA1* (Supplemental Figure S7, C and D).

**Figure 4:**
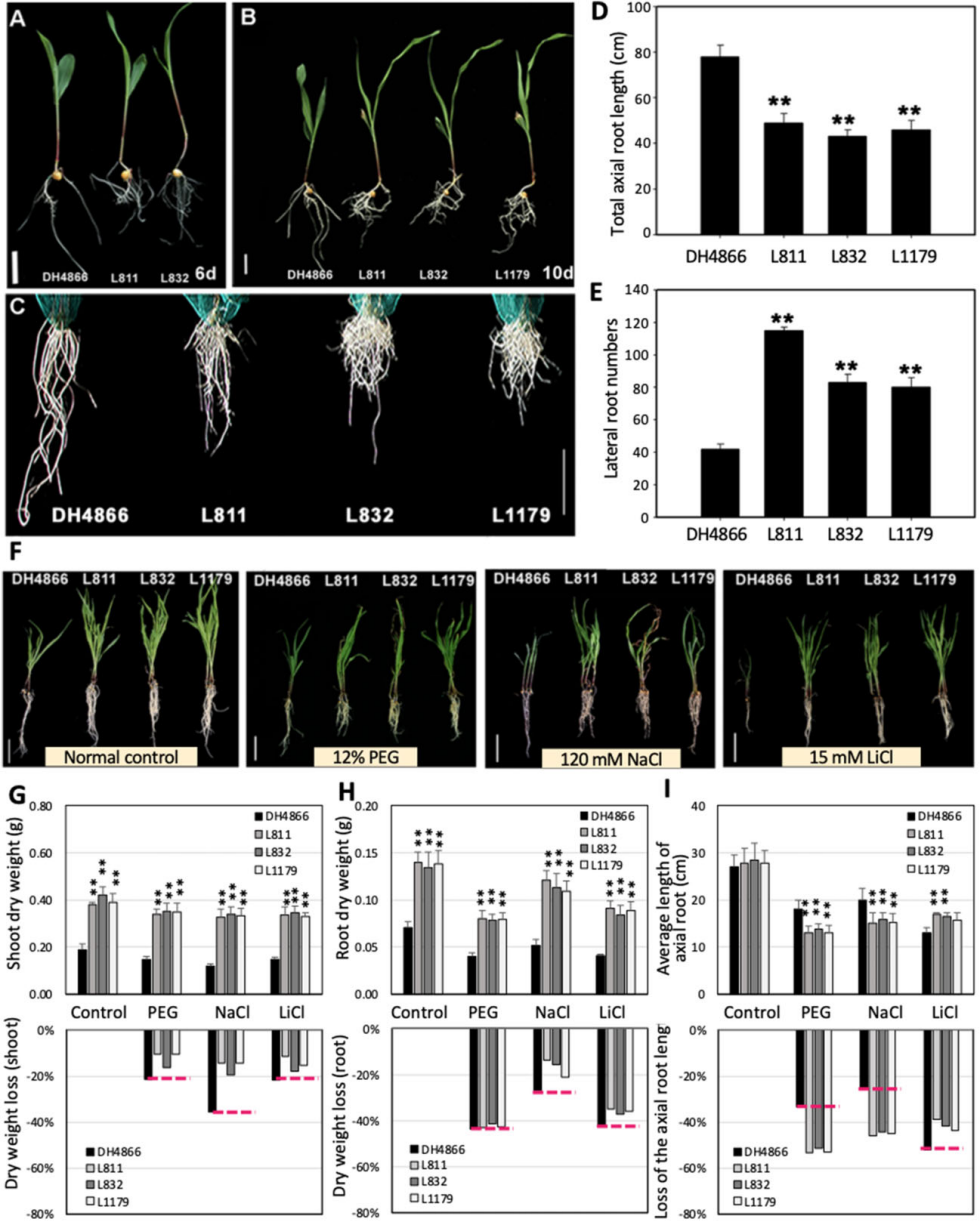
OE of *ZmNF-YA1* improves abiotic stress tolerance in maize. A and B, DH4866 (transgene donor inbred line) and *ZmNF-YA1* OE lines hydroponically cultured for 6 d and 10 d. C, Root phenotypes of DH4866 and *ZmNF-YA1* OE lines hydroponically cultured for 10 d. D and E, Total axial root length and lateral root numbers of DH4866 and *ZmNF-YA1* OE lines hydroponically cultured for 10 d. F, Eleven-day-old hydroponically cultured maize plants of DH4866 and OE lines under normal conditions (Normal Control) and treated with 12% PEG_6000_, 120 mM NaCl, or 15 mM LiCl for 10 d. The images of panels A–C, and F were digitally extracted for comparison. G–I, Shoot dry weight, root dry weight, and axial root length of DH4866 and *ZmNF-YA1* OE lines under different conditions and the loss led by stresses. All values are means ± SD of three independent experiments (each with five seedlings per line). *bar* = 5 cm. The asterisks indicate a significant difference at *0.01<P≤0.05, **P≤0.01 by a *t* test.

To address the effect of ZmNF-YA1 on abiotic stress tolerance in maize, the phenotypes of the transgene donor inbred line DH4866 and ZmNF-YA1 OE plants under different abiotic stresses were analyzed. Analysis of hydroponically cultured plants showed no obvious shoot growth differences of *ZmNF-YA1* OE plants at 6 d after germination (DAG), but the root length was shortened and there were more lateral roots (Figure 4A). The *ZmNF-YA1* OE lines had a substantially higher plant height than DH4866 at 10 DAG due to their enhanced growth rate (Figure 4, B and C). The axial root length was decreased and the lateral root number increased in *ZmNF-YA1* OE plants (Figure 4, D and E). These results suggested that *ZmNF-YA1* played an important role in aerial organ growth and root development. When the 11-d-old hydroponically cultured maize plants were treated with 12% PEG, 120 mM NaCl, or 15 mM LiCl for 10 d, the stresses retarded maize shoot and root growth, and the axial root growth of all seedlings were significantly inhibited, but the responses to stresses were significantly different among the genotypes (Figure 4, F–I), following PEG, NaCl, and LiCl treatment; however, the inhibition was less for *ZmNF-YA1* OE than DH4866 plants (Figure 4, G–I). For example, PEG treatment decreased the shoot biomass of DH4866 by 23% and of *ZmNF-YA1* OE plants by 9%–13% compared with the control under normal conditions. The same trends were observed for the NaCl and LiCl treatments (Figure 4, G–I). By contrast, under normal conditions, the Z*mNF-YA1* OE plants had a larger aerial organ size and a more developed root system with a greater shoot and root biomass than DH4866 (Figure 4F). Therefore, *ZmNF-YA1* OE plants had improved tolerance to abiotic stresses in the vegetative growth stage.

To elucidate the functions of *ZmNF-YA1* in response to drought stress at the flowering stage, maize plants at the V12 stage were subjected to water stress. Under normal conditions, the *ZmNF-YA1* OE lines had a substantially higher plant height than DH4866 at the V12 stage (Supplemental Figure S8A). When *ZmNF-YA1* OE and DH4866 plants at the pre-flowering stage were exposed to drought stress treatment for 12 d, most of the DH4866 leaves appeared chlorotic and wilted; the lower leaves of *ZmNF-YA1* OE plants were withered, while the upper leaves of OE plants remained vigorous (Supplemental Figure S8B). *ZmNF-YA1* OE plants exhibited more root branches and more wheels of brace root than DH4866 after stress treatment (Supplemental Figure S8C). By contrast, drought stress resulted in decreased relative water content and increased ion leakage and malondialdehyde (MDA) content in all plants, and *ZmNF-YA1* OE plants presented significantly higher levels of relative water content and lower levels of ion leakage and MDA content than DH4866. For instance, compared with 78.46% relative water content, 52.97% ion leakage, and 12.94 μmol g^−1^ MDA content in the leaves of DH4866, *ZmNF-YA1* OE plants maintained 83.76%–86.03% relative water content, 37.92%–43.85% ion leakage, and 7.51–8.52 μmol g^−1^ MDA content at 8 d after drought stress (Supplemental Figure S8, D–F). These phenotypic and physiological differences suggested that OE of *ZmNF-YA1* improved tolerance to drought stress.

### Transcriptome analysis of *ZmNF-YA1*-regulated genes under drought conditions

To further examine the role of *ZmNF-YA1* in the drought stress response, the mRNA deep sequencing (GEO Submission [GSE137780]) was performed. Wild-type (W22) and *zmnf-ya1* (m67) plants were grown under well-watered conditions until the V4 stage, and then half of them were exposed to drought stress treatment. We monitored the soil moisture content and electrolyte leakage of leaf cells during the drought stress treatment (Supplemental Figure S9). When exposed to drought stress for 5 d, the top fully expanded leaf of *zmnf-ya1* mutant and W22 plants from normal control and drought stress treatment plants were harvested for RNAseq analysis (Supplemental Tables S1–S3). Principal component analysis showed obvious differences between the *zmnf-ya1* mutant and W22 under normal conditions, whereas when subjected to drought stress treatment, there were fewer differences between the genotypes (Supplemental Figure S10A). When the differentially expressed genes (DEGs) were calculated (absolute log2 fold value > 1 and adjusted *P* <0.05), the *zmnf-ya1* mutant had fewer DEGs (4802 DEGs, with 2,158 upregulated and 2,644 downregulated) compared to W22 (6,814 DEGs, with 3,762 upregulated and 3,052 downregulated) in response to drought stress. And a higher proportion of drought stress repressed DEGs (55.06%) in the *zmnf-ya1* mutant than the 44.79% in W22, whereas the upregulated DEGs showed a reverse change trend (Supplemental Figure S10B and Tables S2–S3). Under normal conditions, 1,225 DEGs had higher expression levels in the *zmnf-ya1* mutant than W22, and 1,101 DEGs had lower expression levels in the *zmnf-ya1* mutant than W22. And when subjected to drought stress treatment, fewer DEGs were present between the genotypes (Supplemental Figure S10C and Tables S2 and S3).

**Figure 5:**
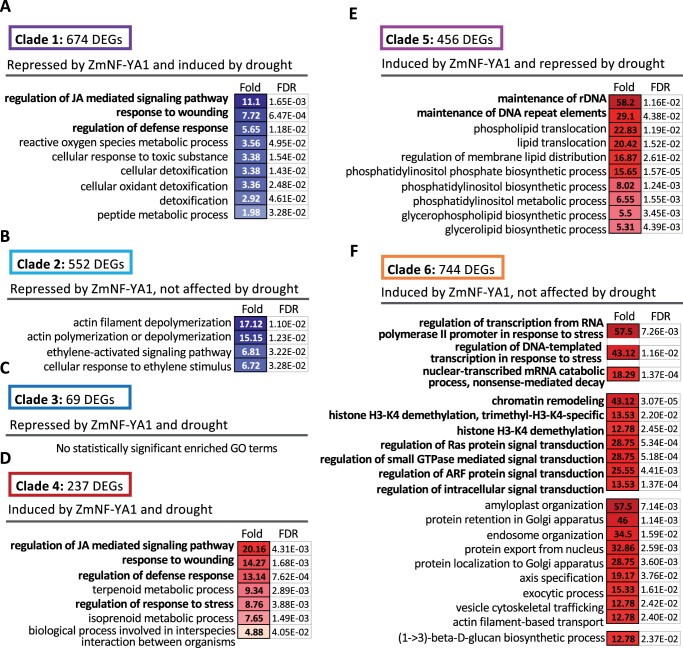
Transcriptome analysis of the *zmnf-ya1* mutant and WT under normal and drought stress. A–C, Significantly enriched GO terms of the Clades 1–3 DEGs repressed by ZmNF-YA1 identified in Supplemental Figure S10D. DEGs in Clades 1–3 were upregulated DEGs in the *zmnf-ya1* mutant (i.e. repressed by ZmNF-YA1) and they could be further grouped into Clades 1–3 based on their response to drought stress. D–F, Significantly enriched GO terms of the Clades 4–6 DEGs induced by ZmNF-YA1 identified in Supplemental Figure S10E. DEGs in Clades 4–6 were downregulated DEGs in the *zmnf-ya1* mutant (i.e. induced by ZmNF-YA1) and they could be further grouped into Clades 4–6 based on their response to drought stress. Values indicate the fold enriched and the FDR values for the corresponding GO terms. The color intensity indicates the enrichment degree. All the GO enrichment analysis results of the six clades DEGs identified in Supplemental Figures S10D and S10E were list in Supplemental Table S4.

Based on the expression patterns, the DEGs associated with both drought and ZmNF-YA1 regulation can be grouped into six clades (Supplemental Figure S10, D and E). To test which clade contributes more to the different growth and stress response of the *zmnf-ya1* mutant and their WT, we performed a Gene Ontology (GO) enrichment analysis of the six clades DEGs (Figure 5 and Supplemental Table S4). In Clade 4, which was induced by ZmNF-YA1 (lower level in *zmnf-ya1* compared to W22) and drought stress, the GO terms of regulation of jasmonic acid (JA) mediated signaling pathway, response to wounding, regulation of defense response and regulation of stress response were on the top and highly enriched (Figure 5D). In Clade 6, which was induced by ZmNF-YA1 alone, GO terms of genes transcription regulation, histone modification and chromatin remodeling were highly enriched (Figure 5F). Compared with the ZmNF-YA1-induced genes, the ZmNF-YA1-repressed genes were involved in stress response and metabolic process, and the enriched fold values were less than that in Clades 4–6 (Figure 5 and Supplemental Table S4). The expression of the key DEGs involved in hormone signaling, transcriptional regulation, and stress response was compared in the *zmnf-ya1* mutant and W22 under both normal and drought stress conditions (Supplemental Figure S11 and Table S5). For the plant hormone signaling, JA signaling pathway was affected more by the abolished *ZmNF-YA1* expression (Figure S11). The genes coding for TFs myelocytomatosis (MYC) 4 (bHLH transcriptional regulator, activate JA-responses), three jasmonate-zim-domain protein (JAZ, also named TIFY) 2, JAZ10 and JAZ11 were induced by drought stress in W22; however, in the *zmnf-ya1* mutant, those JA signaling components failed to increase in response to drought stress. The biosynthesis of JA and ABA were also altered in the *zmnf-ya1* mutant, the coding genes for jasmonate-resistant 1 (JAR1), nine-cis-epoxycarotenoid dioxygenase 9 (NCED9) and two gibberellin 2 oxidase 1 (GA2OX1) shows the same trends, i.e. not sufficiently induced by drought in the *zmnf-ya1* mutant. JAR1 catalyzes the synthesis of jasmonates-AA conjugates by adenylation, the NCED9 catalyzes the first step of ABA biosynthesis from carotenoids, and GA2OX1 converts GA9/GA20 to GA51/GA29 and GA4/GA1 to GA34/GA8. The altered ABA, JA and GA metabolism in the *zmnf-ya1* mutant, especially under drought stress conditions, could be the major reason for the stress tolerance change of the *zmnf-ya1* mutant plants. The bHLH, MYB, NAC, CBF, WRKY TF (Figure S11B), and key stress response factors (Figure S11C) coding genes involved in stress response and growth regulation showed the same changes as the plant hormone signaling genes above. They may transduce/amplify the ZmNF-YA1-mediated early transcription regulation events (both by hormone signaling pathway and chromatin remodeling) and contribute to the stress response in maize.

### Target gene analysis of the ZmNF-YA1

**Figure 6:**
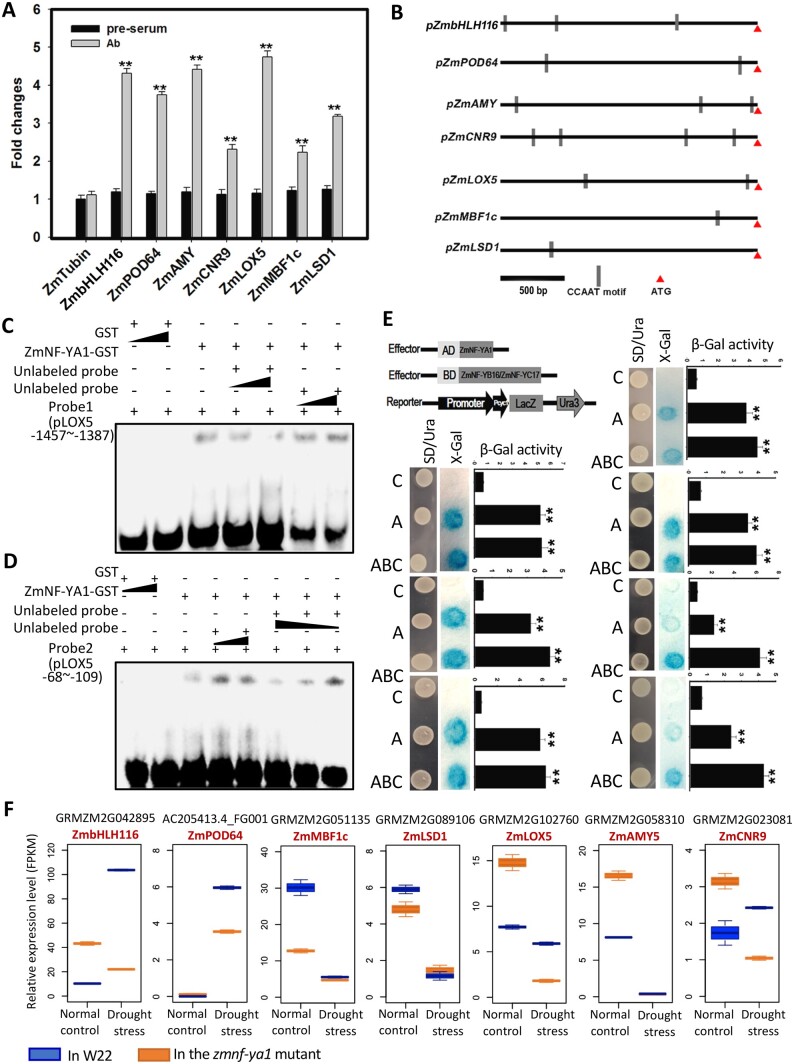
Validation of candidate downstream genes. A Chip-qPCR analysis of the seven candidate downstream genes. B, Distribution of the CCAAT motif on the promoters of candidate downstream genes. C, EMSA showing that ZmNF-YA1 binds to the CCAAT motif at −1,457∼−1,387 bp of the *ZmLOX5* promoter. D, EMSA showing that ZmNF-YA1 binds to the CCAAT motif at −68∼−109 bp of the *ZmLOX5* promoter. E, Yeast one-hybrid analysis of seven candidate downstream genes. C, Control; A, co-transformed with ZmNF-YA1; ABC, co-transformed with ZmNF-YA1, ZmNF-YB16 and ZmNF-YC17. F, Relative expression level boxplot of the seven candidate downstream genes. The blue boxes indicate the expression levels in W22 and the orange boxes indicate the value from the *zmnf-ya1*. In each box, different lines represent the minimum, maximum, median, first quartile, and three quartiles of the dataset.

To test if ZmNF-YA1 directly binds and regulates the expression of the downstream genes, Chromatin immunoprecipitation qPCR (ChIP-qPCR) with the monoclonal antibody of GFP was used to investigate the downstream target genes of *ZmNF-YA1* with *ZmNF-YA1-GFP* transgenic plants. Promoter regions of seven DEGs were selected, and comparisons of ChIP samples with clear immune-precipitation enrichment (2.38–4.75 fold) to mock ChIP were performed (Figure 6A). NF-Y complex recognizes and binds with high affinity and sequence specificity to the cis-acting element CCAAT motif, which is frequently observed in the eukaryotic promoters (Xing et al., 1993). The seven genes have one or more CCAAT motifs in their promoter regions (Figure 6B). To verify the binding between ZmNF-YA1 and the CCAAT motif, an electrophoretic mobility shift assay (EMSA) was adopted. As shown in Figure 6, C and D, ZmNF-YA1 bound to the two core sequences containing the CCAAT motif in the *ZmLOX5* promoter (−1,457∼−1,387; −68∼−109). Then, one-hybrid yeast system assays in the YM4271 strain were conducted to test the binding between ZmNF-YA1 (Figure 6E, co-transformed with ZmNF-YA1 (A)) or the ZmNF-YA1-YB16-YC17 complex (Figure 6E, co-transformed with ZmNF-YA1, YB16, and YC17 (ABC)) and the target genes. After recombining the promoter regions into the pLacZi vector and introducing it into the yeast, β-galactosidase activity was validated by X-gal staining and measured using O-Nitrophenyl β-D-galactopyranoside (ONPG) as substrate. The results showed that ZmNF-YA1 could bind to the target promoter regions, and those co-transformed with the three NF-Ys usually had higher binding activity (Figure 6E). According to the RNA-seq results, a boxplot of the relative expression levels of these seven genes was generated (Figure 6F). The test seven genes had substantially different expression levels in the *zmnf-ya1* mutant compared with W22 at least under one condition. The expression of *ZmMBF1c* (multiprotein bridging factor 1, transcription coactivator, involved in various stresses response), and *ZmLSD1* (zinc finger protein, negatively regulates the basal defense and cell death pathway) were repressed by drought and ZmNF-YA1 works as an activator of the expression of those two genes. *ZmbHLH116* (bHLH TF) and *ZmPOD64* (peroxidase 64) were induced by drought stress, and these two genes were not sufficiently induced in the *zmnf-ya1* mutant under drought stress conditions. For *ZmAMY5* (β-amylase 5), ZmNF-YA1 works as a transcriptional repressor. And for *ZmLOX5* (lipoxygenase 5) and *ZmCNR9* (cell number regulate 9), complex expression changes were observed. The expression patterns of those genes in plants are more complex than those tested in yeast. And the functional redundancy, complementary, and combination of the NF-Y subunits lead to a much more complex and dynamic transcriptional regulation of the downstream genes under normal and stress conditions in plants. Considering the proportion of the up- (52.67%) and downregulated (47.33%) DEGs identified in the comparison of the *zmnf-ya1* mutant compared with W22, ZmNF-YA1 may bind and work as activator/repressor to direct downstream genes.

### Comparison of ZmNF-YB16- and ZmNF-YA1-regulated genes

Both ZmNF-YA1 and ZmNF-YB16 improved drought stress tolerance in maize. To further clarify whether ZmNF-YB16 and ZmNF-YA1 had the same (overlapping) or similar biological functions, we performed a comparative transcriptome analysis by using the DEGs identified in this paper (*zmnf-ya1* vs. W22) and the DEGs ZmNF-YB16 regulated from our previous result (ZmNF-YB16 OE vs. B104 (Wang et al., 2018). In total, 315 DEGs were significantly changed in both *ZmNF-YB16* OE and *zmnf-ya1* under at least one condition (Supplemental Figure S12). The K-mean cluster analysis showed that a high proportion of DEGs was under the co-regulation of ZmNF-YA1 and ZmNF-YB16 (Supplemental Figure S12C), and those DEGs could be further grouped into two major groups. Group 1 (blue line boxed) DEGs were repressed by the ZmNF-YA1 and YB16, whereas Group 2 (auburn line boxed) DEGs were activated by the ZmNF-YA1 and ZmNF-YB16. As shown in Supplemental Figure S12D and Tables S6 and S7, genes coding for transcriptional regulation, and stress responses were co-regulated by ZmNF-YA1 and ZmNF-YB16. TFs NAC036, PYL10, MYB4, PLAT1, IAA7, etc. were in the Group I DEGs identified, which may be repressed by ZmNF-YA1 and ZmNF-YB16. By contrast, genes coding for TFs AP2/B3, PHO2, SNRK3.8, MYB43, the stress response factor transducing, HOT1/HSP101, NB-ARC and a eukaryotic translation initiation factor 3E (EIF3E) were the Group II DEGs, which were activated by ZmNF-YA1 and YB16. The comparative transcriptome analysis implied that *ZmNF-YA1* and *ZmNF-YB16* showed similar biological functions in the stress response process but also had differential functions in other biological processes. In addition, a high proportion of DEGs present in ZmNF-YA1- or ZmNF-YB16-regulated genes were found and speculated to be related to the functional diversity of the ZmNF-YA1 and ZmNF-YB16.

## Discussion

### The ZmNF-YA1/YA7-YB16-YC17 heterotrimer identified in maize

The NF-YA, NF-YB, and NF-YC subunits are encoded by one gene in mammals and yeast, respectively, but the number of each subunit has been expanded in plants (Gusmaroli et al., 2001; Gusmaroli et al., 2002). The NF-Y complex formation mechanism has been extensively studied in yeast and mammals. NF-YB and NF-YC form a heterodimer via their histone fold domain, specifically via the helices α2 and α3 (Kim et al., 1996; Zemzoumi et al., 1999; Frontini et al., 2004). However, the expanding numbers in plants make it difficult to identify the specific subunit that can form a heterotrimeric complex. Although a number of NF-Y subunits have been characterized in model plants, few have been functionally evaluated in crops. Recent work showed that the NF-YB1-YC12-bHLH144 complex directly activates the *Waxy* gene and regulates grain quality in rice (Bello et al., 2019). However, no NF-Y complex has been identified in maize until now. This study demonstrated that ZmNF-YB16 and ZmNF-YC17 could form a heterodimer in the cytoplasm and then form a heterotrimeric complex with ZmNF-YA1 or ZmNF-YA7 in the nucleus by Yeast-3-Hybrdazation, CoIP and BiFC assay (Figures 1 and 2). It can be presumed that the ZmNF-YA1-YB16-YC17 complex formation mechanism is conserved just like in yeast and animals (Romier et al., 2003; Frontini et al., 2004). However, no rice NF-YAs that interacted with the NF-YB1-YC12 heterodimer or NF-YA8 interacted with NF-YB9 without NF-YC family members have been reported (Zhiguo et al., 2018; Bello et al., 2019), suggesting that not all the NF-Y family members follow this principle in yeast and animals. In rice, NF-YB1, NF-YB9, NF-YC11, and NF-YC12 have no transcriptional activation, in contrast to NF-YC8, NF-YC9, and NF-YC10 (Zhiguo et al., 2018). In this study, ZmNF-YB16 interacted with ZmNF-YC17 according to the histone-like domain (Figure 2B), and ZmNF-YB16 and ZmNF-YC17 both had transcriptional activation activity (Figures 1A and 2A), although the region of activity varies. ZmNF-YB16 and ZmNF-YC17 were mostly located in the cytoplasm under normal conditions. Following osmotic stress, they moved into the nucleus to form a heterotrimer with ZmNF-YA1 or ZmNF-YA7. The ZmNF-YA1 heterotrimer could bind to the probe containing a CCAAT motif in the promoter to regulate the expression of target genes (Figures 2, C–E and 6, C–E). Based on the upregulated expression of *ZmNF-YA1* and *ZmNF-YB16* in response to osmotic stress, the amount of ZmNF-YA1/YA7-YB16-YC17 heterotrimers would be increased under osmotic stress conditions. Thus, it was concluded that the ZmNF-Ys subunits played a role in the heterotrimer form in maize, as observed in yeast and mammals, and the heterotrimer had an important role in the abiotic stress tolerance of maize, although the function of each subunit in maize was specific or similar.

### ZmNF-YA1 plays an important role in the regulation of plant growth and abiotic stress tolerance

Functional analyses expounded that plant NF-Ys have multiple roles that regulate the stress response, embryo development, chloroplast biogenesis, root growth, and flowering time (PetroNi et al., 2013). By contrast, the function of maize NF-Ys is much less understood than *Arabidopsis*. Most works remain at the level of gene cloning, expression analysis, and structural characterization. The biological functions, upstream regulators, and downstream targets of NF-Ys are not well understood. *ZmNF-YB2, ZmNF-YB16, ZmNF-YC8*, and *ZmNF-YC2* play important roles in maize stress tolerance or endosperm development (Nelson et al., 2007; Mei et al., 2015; Wang et al., 2018). In this work, we screened the potential interactors with ZmNF-YB16 by the yeast two-hybrid method and confirmed the interaction of the ZmNF-YA1 to ZmNF-YB16-YC17 and ZmNF-YA7 to ZmNF-YB16-YC17. The ZmNF-YA7 (also named ZmNF-YA3)-CONSTANS-like complex has dual functions in the stress response and photoperiod-dependent flowering (Su et al., 2018). These results implied that ZmNF-YA7 could interact with different proteins, including NF-Ys or other TF family members, and these complexes might play various roles in maize biological processes. Being the DNA binding subunit of the heterocomplex, *ZmNF-YA1* OE improved abiotic stress tolerance at the seedling and flowering stage of maize, and it decreased the root length and increased the lateral root number. The *zmnf-ya1* mutant lines showed the opposite phenotypes (Figures 3 and 4). Root architecture has an important role in minimizing the effects of stress on plants, with root proliferation occurring in soil patches with the highest concentration of nutrients and water and avoiding dry or saline patches (Galvan-Ampudia et al., 2013; Karlova et al., 2021). The *ZmNF-YA1* OE lines showed a shorter primary root length and denser lateral roots than WT (Figure 4).

From the comparative transcriptional analysis (*zmnf-ya1* vs. WT, normal and drought stress conditions), GO enrichment analysis showed that Clade 4 (induced by ZmNF-YA1 and drought stress) and Clade 6 (induced by ZmNF-YA1 alone) DEGs contribute to the different growth and stress response of the *zmnf-ya1* mutant and their WT (Figure 5 and Supplemental Table S4). In Clade 4, the GO terms of regulation of JA mediated signaling pathway, response to wounding, regulation of defense response and regulation of stress response were on the top and highly enriched. In Clade 6, which was induced by ZmNF-YA1 alone, GO terms of genes transcription regulation, histone modification and chromatin remodeling were highly enriched. JA signaling pathway was affected more by the abolished *ZmNF-YA1* expression (Supplemental Figure S11). The genes coding for TFs MYC4, three JAZs were induced by drought stress in W22; however, in the *zmnf-ya1* mutant, those JA signaling components failed to increase in response to drought stress. The coding genes for JAR1 (JA metabolism) and NCED9 (ABA biosynthesis) showed the same trends, i.e. insufficiently induced by drought in the *zmnf-ya1* mutant. The altered ABA, JA metabolism and signaling pathway in the *zmnf-ya1* mutant, especially under drought stress conditions, could be the major reason for the stress tolerance change of the *zmnf-ya1* mutant plants. The bHLH, MYB, NAC, CBF, WARKY TF, and key stress response factors (Supplemental Figure S11) may transduce/amplify the ZmNF-YA1-mediated early transcription regulation events and contribute to the stress response and growth regulation in maize. Some of these were selected for binding assay. Moreover, ZmNF-YA1 together with ZmNF-YB16 and ZmNF-YC17 directly regulated the expression levels of the *ZmbHLH116*, *ZmPOD64, ZmAMY, ZmCNR9, ZmLOX5, ZmMBF1c*, and *ZmLSD1* genes by binding to their CCAAT regions in promoters contributed to the enhanced tolerance of abiotic stress in the *ZmNF-YA1* OE maize plants.

### OE of *ZmNF-YA1* or *ZmNF-YB16* produces similar effects on abiotic stress tolerance of maize

Both *ZmNF-YA1* and *ZmNF-YB16* showed high expression levels in leaves at the V5 stage and kernels, and upregulated expression levels in kernels at the five DAP stage after drought stress. *ZmNF-YA1* was also highly expressed in roots at the V5 stage. OE of *ZmNF-YA1* improved drought stress tolerance of maize plants, and OE of *ZmNF-YB16* improved drought stress tolerance and grain yield by enhancing photosynthesis and antioxidant capacity and the ER stress response in maize (Wang et al., 2018). In *ZmNF-YA1* OE lines, one antioxidant peroxidase-coding gene (ZmPOD64) was directly regulated by ZmNF-YA1 (Figure 6). Moreover, the lateral root systems of the *ZmNF-YB16* OE and *ZmNF-YA1* OE lines were more developed than WT, but not all the phenotypes coincided exactly in the *ZmNF-YA1* OE and *ZmNF-YB16* OE lines, such as the inconsistent changes in primary root length. In rice, each member of OsNF-YB1-YC12-bHLH144 exhibits a similar phenotype in seed development (Bello et al., 2019). Transcriptome comparisons are adopted to compare the similarity and difference of ZmNF-YA1- and ZmNF-YB16-regulated genes and processes.

According to the RNA-sequencing analysis, 315 common DEGs regulated by ZmNF-YA1 and ZmNF-YB16 could be further grouped into two major groups. DEGs both repressed (Group 1, blue line boxed in Supplemental Figure S12C) and activated (Group 2, auburn line boxed in Supplemental Figure S12C) DEGs by ZmNF-YA1 and ZmNF-YB16 were found. TFs NAC036, PYL10, MYB4, PLAT1, IAA7 in the Group I DEGs may be repressed by ZmNF-YA1 and ZmNF-YB16. AP2/B3, PHO2, SNRK3.8, MYB43, HOT1/HSP101, NB-ARC and EIF3E activated by ZmNF-YA1 and ZmNF-YB16 were identified. A high proportion of DEGs only present in ZmNF-YA1- or ZmNF-YB16-regulated genes were found and speculated to be related to the functional diversity of the ZmNF-YA1 and ZmNF-YB16. Phenotypic analysis suggested that ZmNF-YA1 and ZmNF-YB16 positively regulated maize plant growth and root development. And both the enhanced stress tolerance ability and developed root system in the OE plants increased the tolerance to stresses such as drought stress in maize. Those processes were at least partly through the ZmNF-YA1-YB16-YC17 complex because they were found to form a heterotrimer by binding assay.

Taken together, ZmNF-YA1-YB16-YC17 might be one type of the complex in maize, and other types of complexes consisting of different NF-Y members might also play a role and provide the diversity of functions in the development and stress response processes of plants. *ZmNF-YB16* has been reported to play important roles in endosperm development because of its direct regulation by *Opaque2* (Li et al., 2015). Additionally, as a target gene of ZmbZIP4, *ZmNF-YA1* has functions in ABA synthesis and the salt stress response (Ma et al., 2018). The diversity of ZmNF-Ys and their partners support this assumption.

**Figure 7:**
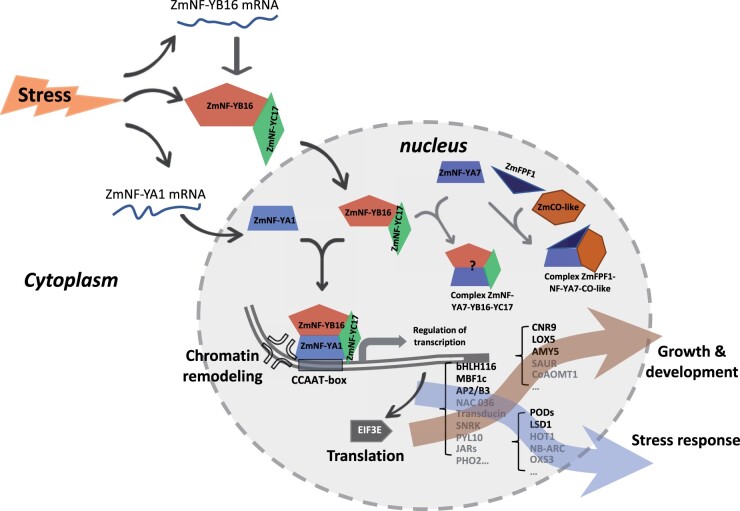
Proposed models of the possible roles of the ZmNF-Y complex in maize development and the stress response. ZmNF-YB16 interacts with ZmNF-YC17 in the cytoplasm forming a dimer, and upon exposure to abiotic stress, the dimer enters the nucleus and binds to ZmNF-YA1 or ZmNF-YA7, forming the ZmNF-YA1-YB16-YC17 or ZmNF-YA7-YB16-YC17 heterotrimer. The expression of *ZmNF-YB16* and *ZmNF-YA1* is induced by stress treatment, whereas *ZmNF-YC17* expression is not. OE of *ZmNF-YA1* and *ZmNF-YB16* significantly improves abiotic stress tolerance and modifies plant growth and development. The downstream targets of the ZmNF-YA1-YB16-YC17 and other ZmNF-YA1-related transcriptional regulation complexes contribute to changed abiotic stress tolerance and plant growth and development in maize. These processes include but are not limited to transcriptional regulation of the ZmNF-YA1 complex through both the CCAAT-box and chromatin remodeling. The target genes coding for TFs, stress response genes, growth and development regulation and translation regulation genes are changed and further contribute to the stress tolerance and growth regulation in maize. Some of these were selected and confirmed as direct targets of the ZmNF-YA1 complex such as *ZmbHLH116*, *ZmPOD64*, *ZmAMY*, *ZmLOX5*, *ZmCNR9*, *ZmMBF1c*, and *ZmLSD1*. ZmNF-YA7 is suggested to interact with other members and play important roles in flowering regulation and stress response.

As summarized in Figure 7, it is concluded that in maize cells, the expression of *ZmNF-YB16* and *ZmNF-YA1* were induced by stress treatment, whereas *ZmNF-YC17* was not. ZmNF-YB16 interacted with ZmNF-YC17 in the cytoplasm to form a dimer, and in response to abiotic stress, entered the nucleus to bind ZmNF-YA1 or ZmNF-YA7 to form ZmNF-YA1-YB16-YC17 or ZmNF-YA7-YB16-YC17 heterotrimer. OE of *ZmNF-YA1* substantially improved abiotic stress tolerance and modified plant growth and development. Additionally, ZmNF-YA1 regulated the transcription of target genes coding for TFs, key factors in stress response and growth, with the latter playing important roles in the stress response and plant development of maize. Those processes include but are not limited to the transcriptional regulation of the ZmNF-YA1 complex through both the CCAAT-box and chromatin remodeling. The ZmNF-YB16-YC17 dimers could form complexes with different subunits, and those complexes might have preferential functions under certain conditions. It will be interesting to understand whether the ZmNF-YB16/-YC17 present in the cytoplasm in the absence of stress indicates an absence of transcriptional regulation, the presence of other functions or no functions.

## Materials and methods

### Plant materials

Maize (*Z. mays*) *zmnf-ya1* mutants carrying a Mu insertion in GRMZM2G000686 were obtained from the UniformMu population, UFMu-06468 and UFMu-02216, which are homozygous lines designated as m67 and m83 in the inbred line W22 background, respectively, and produced by self-pollination for three generations. *ZmNF-YA1* OE plants produced by *A. tumefaciens*-mediated shoot-tip transformation were from an elite inbred line DH4866 of maize. Detail of plants transformation, screen, PCR, RT-qPCR, and southern blot analysis of the OE lines and the mutant lines were in Supplemental Material and Methods. The species used for the BiFC assays was *N. benthamiana* L.

### Bioinformatics analysis

AA sequence alignment of 14 ZmNF-YA proteins, 10 AtNF-YA proteins, and 11 OsNF-YA proteins were performed by downloading data from the Plant Transcription Factor Database v2.0 (Center for Bioinformatics, Peking University, China, http://planttfdb.cbi.pku.edu.cn/) and using the CLUSTAL X 2.0 program (Larkin et al., 2007) and GeneDoc 3.2 program (http://nrbsc.org/gfx/genedoc). The phylogenetic tree was generated with the neighbor-joining (NJ) method using MEGA5 software (Tamura et al., 2011). The sequences of ZmNF-YA family members were downloaded from the Gramene website (http://www.gramene.org/).

### Subcellular localization, western-blot, and CoIP analysis

Maize protoplasts and transient expression were prepared following Sheen’s method (Yoo et al., 2007) and used for the subcellular localization analysis of the maize NF-Y TFs. A vector containing NF-Y fused to pUC18-P35S-*GFP* or pUC18-P35S-*mCherrry* vector was transfected into maize protoplasts. After a 12 h incubation in W1 solution (0.4 M mannitol for normal conditions and 0.6 M mannitol for osmotic stress; Yoo et al., 2007), the protoplasts were observed using the laser scanning confocal microscope (LSM-900, Zeiss, Germany). The respective settings (pinhole = 1 AU; master gain = 673 V; digital gain = 1; digital offset = 0; excitation wavelength = 488 nm and detection wavelength = 490–540 nm for GFP, excitation wavelength = 561 nm and detection wavelength = 590–650 nm for mCherry) were used. Laser intensity, pinhole and detection range were unchanged and similar for all pictures.

*Nicotiana benthamiana* protoplasts and transient expression were prepared following Sheen’s method (Yoo et al., 2007) and used for the Co-IP and the western-blot with cytosol and nuclear fraction assay. The corresponding vector containing NF-Y fused to GFP or mCherry was transiently transfected into the protoplasts. The isolation of cytosol and nuclear fraction from protoplast was performed as described in Ryu et al. (2010) except the *N. benthamiana* leaves were used. In brief, the protoplasts were lysed with a lysis buffer and then centrifugation at 3,000 *g* for 10 min. The cytosolic fraction in the supernatant was collected and stored on ice until use. The nuclear pellets were further washed with a resuspension buffer. The proteins in each fraction were subjected to 10% SDS-PAGE, followed by immunodetection. The anti-GFP (HT801, TransGen Biotech, Beijing, China, 1:2,000 dilution), Goat anti-mouse IgG h&L (HRP; SA00002-1, Proteintech, Chicago, USA, 1:5,000 dilution), and immunodetection using BCIP/NBT substrate (PR1100, Solarbio, Beijing, China) were used for the western-blot.

For the CoIP assays, approximately 2 × 10^5^ leaf mesophyll protoplasts were transfected and incubated for 24 h. After harvest, the cells were lysed with 200 μL IP buffer (50 mM Tris-HCl at pH 7.5, 150 mM NaCl, 5 mM EDTA, 1% [v/v] Triton X-100, 0.5 mM DTT, and one table Complete Protease Inhibitor (04693159001, Roche, Germany). A 50 μL aliquot was taken as the input control and the remaining lysate was incubated with antibody (anti-GFP antibody (ab290, Abcam, Cambridge, UK) or anti-mCherry antibody (ab213511, Abcam, Cambridge, UK) for 1 h at 4°C gentle rotation. Protein-A beads (DP501, TransGen Biotech, Beijing, China) was used to purify the antibody-protein complex from the mixture. After incubation, the beads were washed 5 times with IP buffer. Eluted samples and input samples were subjected to immunoblot analysis using conjugated anti-GFP (HT801, TransGen Biotech, Beijing, China, 1:2,000 dilution) or anti-mCherry antibodies (ab213511, Abcam, Cambridge, UK, 1:1,000). The secondary antibodies are Goat anti-mouse IgG h&L (HRP; SA00002-1, Proteintech, Chicago, USA, 1:5,000 dilution) or Goat anti-Rabbit IgG h&L (HRP; SA00002-2, Proteintech, Chicago, USA, 1:5,000 dilution), respectively.

### BiFC assays

For the BiFC assays, full-length *ZmNF-YB16* and *ZmNF-YC17* were cloned into the pSPYCN-35S-*cYFP* and pSPYCN-35S-*nYFP* vectors. Suspensions of Agrobacteria containing pSPYCN-35S-*ZmNF-YB16-cYFP*, pSPYCN-35S-*ZmNF-YC17-nYFP*, pSPYCN-35S-*cYFP*, and pSPYCN-35S-*nYFP* were injected into the lower epidermis of *N. benthamiana* leaves. The transfected plants were kept in the greenhouse for at least 36 h at 22°C. Fluorescent signals were visualized using an LSM-700 laser scanning confocal microscope (LSM-700, Zeiss, Germany). For eYFP signal detection, excitation wavelength (488 nm), and detection wavelength (500–580 nm) were used. Primers used for the constructs are shown in Supplemental Table S8.

### Pull-down assay

For the pull-down assay, the recombinant ZmNF-YC17-GST and ZmNF-YB16-His were purified from bacteria using glutathione beads (17075601, GE Healthcare, UK) and Ni-NTA beads (DP101-01, Transgen, Beijing, China), respectively. Glutathione beads containing 1 µg of GST or GST-ZmNF-YC17 were incubated with 1 µg ZmNF-YB16-His as indicated in pull-down buffer (20 mM Tris-HCl, pH 7.5, 100 mM NaCl, 1 mM EDTA) for 1 h, and washed 5 times with wash buffer (20 mM Tris-HCl, pH 7.5, 300 mM NaCl, 0.1% v/v Nonidet P-40, 1 mM EDTA). Proteins were eluted from the beads by boiling in 50 μL 2× SDS sample buffer and separated on 10% SDS-PAGE gels. Gel blots were analyzed using anti-His and anti-GST antibodies (66005-1-Ig (anti-His) and 66001-2-Ig (anti-GST), Proteintech, Chicago, USA).

### Transcription activation activity assay and yeast two-hybrid screening

The different lengths of ZmNF-YB16 and ZmNF-YC17 were ligated into the *Eco*RI and *Bam*HI sites of pGBKT7-BD. Plasmids were transformed in the yeast reporter strain YRG-2 (Agilent Stratagene, California, USA). The β-galactosidase activities were examined by X-gal staining and measured using the o-nitrophenyl-β-D- galactopyranoside (ONPG) assay (N1127, Sigma-Aldrich, Shanghai, China).

A maize cDNA library was prepared in the pGADT7 vector in MatchMaker^TM^ GAL4 two-hybrid system 3 (Clontech, USA) using RNA isolated from B73 V3 stage seedlings according to the manufacturer’s instructions. Yeast two-hybrid screening and two-hybrid or three-hybrid interaction assays were performed as described in the user manual supplied with the Matchmaker^TM^ GAL4 Two-Hybrid System3 Libraries User Manual (Clontech, USA). Primers used are shown in Supplemental Table S8.

### Binding activity assay for ZmNF-YA1 and ZmNF-YA1/ZmNF-YB16/ZmNF-YC17 in the yeast system

The coding sequences of *ZmNF-YA1*, *ZmNF-YB16*, and *ZmNF-YC17* were obtained by PCR. *ZmNF-YA1* was digested with *Eco*RI/*Bam*HI and then ligated into the pGADT7-AD vector containing the GAL4 AD domain. *ZmNF-YB16* was digested with *Eco*RI/*Bam*HI, and *ZmNF-YC17* with *Bgl*II, and then ligated into the pBridge vector containing the GAL4 BD domain. Promoters of the candidate target genes were cloned into the *Hind*III/*Kpn*I sites of pLacZi. Plasmids were transformed into yeast strain YM4271 (Invitrogen, California, USA) in pairs. β-galactosidase activities were examined by X-gal staining and measured as described in the Yeast Protocols Handbook (Clontech, USA) using ONPG (N1127, Sigma-Aldrich, Shanghai, China) as substrate.

### Stress treatment of maize plants

The phenotype analysis in the seedling stage was performed using the hydroponic culture method (Li et al., 2011). The V3 stage (11-d-old) seedlings were used for stress treatments with or without 12% PEG_6000_, 120 mM NaCl, or 15 mM LiCl in the nutrient solution. After 10 d of dehydration stress, salt stress or ion toxic treatments, the plants were harvested to measure the biomass and morphological parameters. Three independent replicates were assessed, and 10–15 individual plants in each replicate. A drought stress treatment was performed during the reproductive growth stage (V10 stage) was performed by pot cultivation as described by Li et al., (2008a). Morphological, physiological analyses were performed as described in Supplemental material and methods.

### RNA-seq analysis

Two sets of RNA-seq were used in this paper, i.e. the *zmnf-ya1* (m67) vs. W22 (wild-type maize inbred line for mutant line) and *ZmNF-YB16* OE (L187) vs. B104 (wild-type maize inbred line for the transgenic donor). To compare *zmnf-ya1* (m67) vs. W22, maize plants were grown in pots in a greenhouse. The plants were grown under well-watered conditions until the V4 stage, and then half of them were exposed to drought stress treatment. Water loss in the soil was determined using a soil moisture content analyzer (TOP instrument, Zhejiang, China). Electrolyte leakage from leaf cells was used to assess the injured degree of plants. The sampling time points were determined by monitoring the soil moisture content and leaf cell electrolyte leakage. Leaves from three to four plants were pooled for each sample, and two replicates were used in the study.

For the comparison of ZmNF-YB16 OE line vs. B104, data and drought stress treatment are from our previously published papers (Wang et al., 2018; Wang et al., 2019; Details were in Supplemental material and methods and papers cited). In this paper, RNA extraction, cDNA libraries construction, RNA-sequencing, filtering, and mapping to reference genome (maize B73 V3 5b+) were performed by BGI Tech Solutions Co., Ltd (Shenzhen, China), according to standard procedures (details in Supplemental material and methods). The bioinformatics analysis for digital gene expression profiling was performed according to the bioinformatics analysis procedure of BGI Tech. The criteria of false discovery rate (FDR) ≤0.001 and the absolute value of log_2_ ratio ≥1 was used as thresholds to judge the significance of differences in gene expression (data deposited GSE137780). Gene functional annotations were conducted based on MaizeGDB (Version: 284 *Z. mays* release of Phytozome 10; https://www.maizegdb.org). GO enrichment analysis was based on the AGRIGO database (http://bioinfo.cau.edu.cn/agriGO/) and the GENEONTOLOGY (http://geneontology.org).

### ChIP‐qPCR

The construct pCAMBIA1302-P35S-*ZmNF-YA1-GFP* was used for the transformation of the young embryos of maize HiII via a gene gun (Bio-Rad, USA). Plants were produced from transgenic calli. Stable transgenic maize and the anti-GFP antibody (ChIP-grade ab290 from Abcam, Cambridge, UK) were used for the ChIP-qPCR assay. ChIP was performed with the method described originally by Kaufmann et al. (Kaufmann et al., 2010) with minor modifications and ChIP-qPCR were performed. The primers used to amplify the enriched region of the target genes are listed in Supplemental Table S8.

### EMSA

Probes were generated using the EMSA Probe Biotin Labeling Kit (GS008, Beyotime, Shanghai, China). The labeled 40 bp key fragments or excess unlabeled fragments of the promoter were incubated with ZmNF-YA14-GST and biotin-11-dUTP at 25°C for 30 min. The samples were subjected to electrophoresis at 80 V on 10% PAGE gels running with 0.5× TEA buffer for 1.5 h. Then, the generated chemiluminescent signals were recorded on an imaging device according to the methods supplied with the chemiluminescent EMSA kit (GS0009, Beyotime, Shanghai, China).

## Statistical analysis

All data are presented as the mean values of at least three independent sets of experiments. The data are reported as the mean ± SD. Statistical significance was analyzed using the Student’s *t* test with SPSS 16.0 software (IBM, USA).

## Accession numbers

Sequence data from this article can be found in the MaizeGDB data libraries (https://www.maizegdb.org/) under the accession numbers of the genes listed in Supplemental Table S9. The expression data reported are available in the NCBI Gene Expression Omnibus (GEO) database under the GSE series accession number GSE137780.

## Supplemental data

The following materials are available in the online version of this article.

**Supplemental Figure S1.** The original images of pull-down, Co-IP and western-blot analyses used in this paper.

**Supplemental Figure S2.** Phylogenetic analysis of NF-YA family members of maize.

**Supplemental Figure S3.** Multiple sequence alignment of NF-YA family members from maize and rice.

**Supplemental Figure S4.** Organ expression patterns of the genes related to the heterotrimer.

**Supplemental Figure S5.** Expression profiles of the NF-Y complex members under different abiotic stresses.

**Supplemental Figure S6.** *ZmNF-YA1* regulates plant growth in maize.

**Supplemental Figure S7.** Molecular characterization of *zmnf-ya1* mutants and *ZmNF-YA1* OE transgenic plants.

**Supplemental Figure S8.** Overexpression of *ZmNF-YA1* confers the drought tolerance of maize plants in the fields.

**Supplemental Figure S9.** Measurement of physiological index of samples for RNA-sequencing.

**Supplemental Figure S10.** Overview of the RNAseq analysis and the six different clades DEGs regulated by ZmNF-YA1.

**Supplemental Figure S11.** Expression of the key DEGs in the comparisons of the *zmnf-ya1* mutant versus W22.

**Supplemental Figure S12.** Comparative transcriptome analysis of ZmNF-YA1- and ZmNF-YB16-regulated genes.

**Supplemental Table S1.** Reads and mapping of the sequence samples for *zmnf-ya1* mutant and W22.

**Supplemental Table S2.** All the DEGs identified in *zmnf-ya1* mutant and W22 plants under normal and drought stress conditions.

**Supplemental Table S3.** Gene relative expression level (FPKM) identified in this paper.

**Supplemental Table S4.** GO enrichment analysis of the six groups DEGs in Figure 5 and Supplemental Figure S10.

**Supplemental Table S5.** Expression of the key DEGs used in Figure 6 and Supplemental Figure S11.

**Supplemental Table S6.** The 315 DEGs co-regulated by ZmNF-YA1 and ZmNF-YB16.

**Supplemental Table S7.** The 32 coregulated DEGs used for heatmap plotting Figure S12.

**Supplemental Table S8.** List of primers used in this research.

**Supplemental Table S9.** Gene accession numbers related to this research.

## Supplementary Material

kiac340_Supplementary_Data

## Notes

Y.Y., B.W., and J.W. performed most of the experiments and analyzed the data; C. H. and D.Z. provided technical assistance; P.L. performed a part of the experiments; Z.L., B.W., and J.Z., designed the experiment and completed the writing.

The author responsible for distribution of materials integral to the findings presented in this article in accordance with the policy described in the Instructions for Authors (https://academic.oup.com/plphys/pages/general-instructions) is: Zhaoxia Li (zhaoxia_1019@126.com).

## References

[kiac340-B1] Alam MM , Tanaka T, Nakamura H, Ichikawa H, Kobayashi K, Yaeno T, Yamaoka N, Shimomoto K, Takayama K, Nishina H, et al (2015) Overexpression of a rice heme activator protein gene (OsHAP2E) confers resistance to pathogens, salinity and drought, and increases photosynthesis and tiller number. Plant Biotechnol J 13: 85–96 25168932 10.1111/pbi.12239

[kiac340-B2] Ballif J , Endo S, Kotani M, MacAdam J, Wu Y (2011) Over-expression of HAP3b enhances primary root elongation in Arabidopsis. Plant Physiol Biochem 49: 579–583 21316979 10.1016/j.plaphy.2011.01.013

[kiac340-B3] Bello BK , Hou Y, Zhao J, Jiao G, Wu Y, Li Z, Wang Y, Tong X, Wang W, Yuan W, et al (2019) NF-YB1-YC12-bHLH144 complex directly activates Wx to regulate grain quality in rice (Oryza sativa L.). Plant Biotechnol J 17: 1222–1235 30552799 10.1111/pbi.13048 PMC6576074

[kiac340-B4] Feng ZJ , He GH, Zheng WJ, Lu PP, Chen M, Gong YM, Ma YZ, Xu ZS (2015) Foxtail millet NF-Y families: Genome-wide survey and evolution analyses identified two functional genes important in abiotic stresses. Front Plant Sci 6: 1149 26734043 10.3389/fpls.2015.01142 PMC4687410

[kiac340-B5] Frontini M , Imbriano C, Manni I, Mantovani R (2004) Cell cycle regulation of NF-YC nuclear localization. Cell Cycle 3: 217–222 14712092

[kiac340-B6] Galvan-Ampudia CS , Julkowska MM, Darwish E, Gandullo J, Korver RA, Brunoud G, Haring MA, Munnik T, Vernoux T, Testerink C (2013) Halotropism is a response of plant roots to avoid a saline environment. Curr Biol 23: 2044–2050 24094855 10.1016/j.cub.2013.08.042

[kiac340-B7] Gnesutta N , Kumimoto RW, Swain S, Chiara M, Siriwardana C, Horner DS, Holt BF, Mantovani R (2017) CONSTANS imparts DNA sequence specificity to the histone fold NF-YB/NF-YC Dimer. Plant Cell 29: 1516–1532 28526714 10.1105/tpc.16.00864 PMC5502446

[kiac340-B8] Gusmaroli G , Tonelli C, Mantovani R (2001) Regulation of the CCAAT-binding NF-Y subunits in Arabidopsis thaliana. Gene 264: 173–185 11250072 10.1016/s0378-1119(01)00323-7

[kiac340-B9] Gusmaroli G , Tonelli C, Mantovani R (2002) Regulation of novel members of the Arabidopsis thaliana CCAAT-binding nuclear factor Y subunits. Gene 283: 41–48 11867211 10.1016/s0378-1119(01)00833-2

[kiac340-B10] Ito Y , Thirumurugan T, Serizawa A, Hiratsu K, Ohme-Takagi M, Kurata N (2011) Aberrant vegetative and reproductive development by overexpression and lethality by silencing of OsHAP3E in rice. Plant Sci 181: 105–110 21683874 10.1016/j.plantsci.2011.04.009

[kiac340-B11] Kahle J , Baake M, Doenecke D, Albig W (2005) Subunits of the heterotrimeric transcription factor NF-Y are imported into the nucleus by distinct pathways involving importin β and importin 13. Mol Cell Biol 25: 5339–5354 15964792 10.1128/MCB.25.13.5339-5354.2005 PMC1157003

[kiac340-B12] Karlova R , Boer D, Hayes S, Testerink C (2021) Root plasticity under abiotic stress. Plant Physiol 187: 1057–1070 34734279 10.1093/plphys/kiab392 PMC8566202

[kiac340-B13] Kaufmann K , Muiño JM, Østerås M, Farinelli L, Krajewski P, Angenent GC (2010) Chromatin immunoprecipitation (ChIP) of plant transcription factors followed by sequencing (ChIP-SEQ) or hybridization to whole genome arrays (ChIP-CHIP). Nat Protoc 5: 457–472 20203663 10.1038/nprot.2009.244

[kiac340-B14] Kim IS , Sinha S, de Crombrugghe B, Maity SN (1996) Determination of functional domains in the C subunit of the CCAAT-binding factor (CBF) necessary for formation of a CBF-DNA complex: CBF-B interacts simultaneously with both the CBF-A and CBF-C subunits to form a heterotrimeric CBF molecule. Mol Cell Biol 16: 4003–4013 8754798 10.1128/mcb.16.8.4003 PMC231396

[kiac340-B15] Laloum T , De Mita S, Gamas P, Baudin M, Niebel A (2013) CCAAT-box binding transcription factors in plants: Y so many? Trends Plant Sci 18: 157–166 22939172 10.1016/j.tplants.2012.07.004

[kiac340-B16] Larkin MA , Blackshields G, Brown NP, Chenna R, Mcgettigan PA, McWilliam H, Valentin F, Wallace IM, Wilm A, Lopez R, et al (2007) Clustal W and Clustal X version 2.0. Bioinformatics 23: 2947–2948 17846036 10.1093/bioinformatics/btm404

[kiac340-B17] Leyva-González MA , Ibarra-Laclette E, Cruz-Ramírez A, Herrera-Estrella L (2012) Functional and transcriptome analysis reveals an acclimatization strategy for abiotic stress tolerance mediated by Arabidopsis NF-YA family members. PLoS One 7: e48138 23118940 10.1371/journal.pone.0048138 PMC3485258

[kiac340-B18] Li B , Wei A, Song C, Li N, Zhang J (2008a) Heterologous expression of the TsVP gene improves the drought resistance of maize. Plant Biotechnol J 6: 146–159 17999658 10.1111/j.1467-7652.2007.00301.x

[kiac340-B19] Li C , Qiao Z, Qi W, Wang Q, Yuan Y, Yang X, Tang Y, Mei B, Lv Y, Zhao H, et al (2015) Genome-wide characterization of cis-acting DNA targets reveals the transcriptional regulatory framework of Opaque2 in maize. Plant Cell 27: 532–545 25691733 10.1105/tpc.114.134858 PMC4558662

[kiac340-B20] Li WX , Oono Y, Zhu J, He XJ, Wu JM, Iida K, Lu XY, Cui X, Jin H, Zhu JK (2008b) The Arabidopsis NFYA5 transcription factor is regulated transcriptionally and posttranscriptionally to promote drought resistance. Plant Cell 20: 2238–2251 18682547 10.1105/tpc.108.059444 PMC2553615

[kiac340-B21] Li YJ , Fang Y, Fu YR, Huang JG, Wu CA, Zheng CC (2013) NFYA1 Is Involved in Regulation of Postgermination Growth Arrest Under Salt Stress in Arabidopsis. PLoS One 8: e61289 23637805 10.1371/journal.pone.0061289 PMC3634844

[kiac340-B22] Li Z , Gao Q, Liu Y, He C, Zhang X, Zhang J (2011) Overexpression of transcription factor ZmPTF1 improves low phosphate tolerance of maize by regulating carbon metabolism and root growth. Planta 233: 1129–1143 21312041 10.1007/s00425-011-1368-1

[kiac340-B23] Liu JX , Howell SH (2010) bZIP28 and NF-Y transcription factors are activated by ER stress and assemble into a transcriptional complex to regulate stress response genes in Arabidopsis. Plant Cell 22: 782–796 20207753 10.1105/tpc.109.072173 PMC2861475

[kiac340-B24] Luan M , Xu M, Lu Y, Zhang L, Fan Y, Wang L (2015) Expression of zma-miR169 miRNAs and their target ZmNF-YA genes in response to abiotic stress in maize leaves. Gene 555: 178–185 25445264 10.1016/j.gene.2014.11.001

[kiac340-B25] Luan M , Xu M, Lu Y, Zhang Q, Zhang L, Zhang C, Fan Y, Lang Z, Wang L (2014) Family-wide survey of miR169s and NF-YAs and their expression profiles response to abiotic stress in maize roots. PLoS One 9: 91369 10.1371/journal.pone.0091369 PMC3954700 24633051

[kiac340-B26] Ma H , Liu C, Li Z, Ran Q, Xie G, Wang B, Fang S, Chu J, Zhang J (2018) ZmbZIP4 contributes to stress resistance in maize by regulating ABA synthesis and root development. Plant Physiol 178: 753–770 30126870 10.1104/pp.18.00436 PMC6181033

[kiac340-B27] Manimaran P , Venkata Reddy S, Moin M, Raghurami Reddy M, Yugandhar P, Mohanraj SS, Balachandran SM, Kirti PB (2017) Activation-tagging in indica rice identifies a novel transcription factor subunit, NF-YC13 associated with salt tolerance. Sci Rep 7: 9341 28839256 10.1038/s41598-017-10022-9 PMC5570948

[kiac340-B28] Mei X , Liu C, Yu T, Liu X, Xu D, Wang J, Wang G, Cai Y (2015) Identification and characterization of paternal-preferentially expressed gene NF-YC8 in maize endosperm. Mol Genet Genomics 290: 1819–1831 25851237 10.1007/s00438-015-1043-5

[kiac340-B29] Nelson DE , Repetti PP, Adams TR, Creelman RA, Wu J, Warner DC, Anstrom DC, Bensen RJ, Castiglioni PP, Donnarummo MG, et al (2007) Plant nuclear factor Y (NF-Y) B subunits confer drought tolerance and lead to improved corn yields on water-limited acres. Proc Natl Acad Sci USA 104: 16450–16455 17923671 10.1073/pnas.0707193104 PMC2034233

[kiac340-B30] Ni Z , Hu Z, Jiang Q, Zhang H (2013) GmNFYA3, a target gene of miR169, is a positive regulator of plant tolerance to drought stress. Plant Mol Biol 82: 113–129 23483290 10.1007/s11103-013-0040-5

[kiac340-B31] Petroni K , Kumimoto RW, Gnesutta N, Calvenzani V, Fornari M, Tonelli C, Holt BF, Mantovani R (2013) The promiscuous life of plant NUCLEAR FACTOR Y transcription factors. Plant Cell 24: 4777–4792 10.1105/tpc.112.105734 PMC3556957 23275578

[kiac340-B32] Qu B , He X, Wang J, Zhao Y, Teng W, Shao A, Zhao X, Ma W, Wang J, Li B, et al (2015) A wheat CCAAT box-binding transcription factor increases the grain yield of wheat with less fertilizer input. Plant Physiol 167: 411–423 25489021 10.1104/pp.114.246959 PMC4326744

[kiac340-B33] Romier C , Cocchiarella F, Mantovani R, Moras D (2003) The NF-YB/NF-YC structure gives insight into DNA binding and transcription regulation by CCAAT factor NF-Y. J Biol Chem 278: 1336–1345 12401788 10.1074/jbc.M209635200

[kiac340-B34] Ryu H , Kim K, Cho H, Hwang I (2010) Predominant actions of cytosolic BSU1 and nuclear BIN2 regulate subcellular localization of bes1 in brassinosteroid signaling. Mol Cells 29: 291–296 20387035 10.1007/s10059-010-0034-y

[kiac340-B35] Sato H , Mizoi J, Tanaka H, Maruyama K, Qin F, Osakabe Y, Morimoto K, Ohori T, Kusakabe K, Nagata M, et al (2014) Arabidopsis DPB3-1, A DREB2A interactor, specifically enhances heat stress-induced gene expression by forming a heat stress-specific transcriptional complex with NF-Y subunits. Plant Cell 26: 4954–4973 25490919 10.1105/tpc.114.132928 PMC4311209

[kiac340-B36] Sekhon RS , Lin H, Childs KL, Hansey CN, Robin Buell C, De Leon N, Kaeppler SM (2011) Genome-wide atlas of transcription during maize development. Plant J 66: 553–563 21299659 10.1111/j.1365-313X.2011.04527.x

[kiac340-B37] Siefers N , Dang KK, Kumimoto RW, Bynum IV WE, Tayrose G, Holt BF (2009) Tissue-specific expression patterns of Arabidopsis NF-Y transcription factors suggest potential for extensive combinatorial complexity. Plant Physiol 149: 625–641 19019982 10.1104/pp.108.130591 PMC2633833

[kiac340-B38] Sinha S , Maity SN, Lu J, De Crombrugghe B (1995) Recombinant rat CBF-C, the third subunit of CBF/NFY, allows formation of a protein-DNA complex with CBF-A and CBF-B and with yeast HAP2 and HAP3. Proc Natl Acad Sci USA 92: 1624–1628 7878029 10.1073/pnas.92.5.1624 PMC42572

[kiac340-B39] Sorin C , Declerck M, Christ A, Blein T, Ma L, Lelandais-Brière C, Njo MF, Beeckman T, Crespi M, Hartmann C (2014) A miR169 isoform regulates specific NF-YA targets and root architecture in Arabidopsis. New Phytol 202: 1197–1211 24533947 10.1111/nph.12735

[kiac340-B40] Soyano T , Kouchi H, Hirota A, Hayashi M (2013) NODULE INCEPTION directly targets NF-Y subunit genes to regulate essential processes of root nodule development in lotus japonicus. PLoS Genet 9: e1003352 23555278 10.1371/journal.pgen.1003352 PMC3605141

[kiac340-B41] Stelpflug SC , Sekhon RS, Vaillancourt B, Hirsch CN, Buell CR, Leon N, Kaeppler SM (2016) An expanded maize gene expression atlas based on RNA sequencing and its use to explore root development. Plant Genome10.3835/plantgenome2015.04.0025 27898762

[kiac340-B42] Su H , Cao Y, Ku L, Yao W, Cao Y, Ren Z, Dou D, Wang H, Ren Z, Liu H, et al (2018) Dual functions of ZmNF-YA3 in photoperiod-dependent flowering and abiotic stress responses in maize. J Exp Bot 69: 5177–5189 30137393 10.1093/jxb/ery299

[kiac340-B43] Su H , Chen Z, Dong Y, Ku L, Abou-Elwafa SF, Ren Z, Cao Y, Dou D, Liu Z, Liu H, et al (2021) Identification of ZmNF-YC2 and its regulatory network for maize flowering time. J Exp Bot 72: 7792–7807 34338753 10.1093/jxb/erab364

[kiac340-B44] Tamura K , Peterson D, Peterson N, Stecher G, Nei M, Kumar S (2011) MEGA5: molecular evolutionary genetics analysis using maximum likelihood, evolutionary distance, and maximum parsimony methods. Mol Biol Evol 28: 2731–2739 21546353 10.1093/molbev/msr121 PMC3203626

[kiac340-B45] Wang B , Li Z, Ran Q, Li P, Peng Z, Zhang J (2018) ZmNF-YB16 overexpression improves drought resistance and yield by enhancing photosynthesis and the antioxidant capacity of maize plants. Front Plant Sci 9: 709 29896208 10.3389/fpls.2018.00709 PMC5986874

[kiac340-B46] Wang B , Liu C, Zhang D, He C, Zhang J, Li Z (2019) Effects of maize organ-specific drought stress response on yields from transcriptome analysis. BMC Plant Biol 19: 1–19 31370805 10.1186/s12870-019-1941-5 PMC6676540

[kiac340-B47] Winter D , Vinegar B, Nahal H, Ammar R, Wilson GV, Provart NJ (2007) An “electronic fluorescent pictograph” Browser for exploring and analyzing large-scale biological data sets. PLoS One 2: e718 17684564 10.1371/journal.pone.0000718 PMC1934936

[kiac340-B48] Xing Y , Files JD, Guarente L (1993) Mutations in yeast HAP2/HAP3 define a hybrid CCAAT box binding domain. EMBO J 12: 4647–4655 8223474 10.1002/j.1460-2075.1993.tb06153.x PMC413902

[kiac340-B49] Yoo SD , Cho YH, Sheen J (2007) Arabidopsis mesophyll protoplasts: A versatile cell system for transient gene expression analysis. Nat Protoc 2: 1565–1572 17585298 10.1038/nprot.2007.199

[kiac340-B50] Zemzoumi K , Frontini M, Bellorini M, Mantovani R (1999) NF-Y histone fold α1 helices help impart CCAAT specificity. J Mol Biol 286: 327–337 9973554 10.1006/jmbi.1998.2496

[kiac340-B51] Zhang J-J , Xue H-W (2013) OsLEC1/OsHAP3E participates in the determination of meristem identity in both vegetative and reproductive developments of rice. J Integr Plant Biol 55: 232–249 23230849 10.1111/jipb.12025

[kiac340-B52] Zhang Z , Li X, Zhang C, Zou H, Wu Z (2016) Isolation, structural analysis, and expression characteristics of the maize nuclear factor Y gene families. Biochem Biophys Res Commun 478: 752–758 27498027 10.1016/j.bbrc.2016.08.020

[kiac340-B53] Zhiguo E , Li T, Zhang H, Liu Z, Deng H, Sharma S, Wei X, Wang L, Niu B, Chen C (2018) A group of nuclear factor y transcription factors are sub-functionalized during endosperm development in monocots. J Exp Bot 69: 2495–2510 29514259 10.1093/jxb/ery087 PMC5920288

