# Supplementary material for: Transcription factors ZmNF-YA1 and ZmNF-YB16 regulate plant growth and drought tolerance in maize
Source: Plant Physiol. 2022 Jul 21;190(2):1506–25. doi: 10.1093/plphys/kiac340 (PMC9516732; doi:10.1093/plphys/kiac340)
Supplement: kiac340_Supplementary_Data [file kiac340_supplementary_data.zip › Supplemental Figures 112 and Supplemental Material and methods.pdf]

Supplemental Figures S1-12 and supplemental material and methods

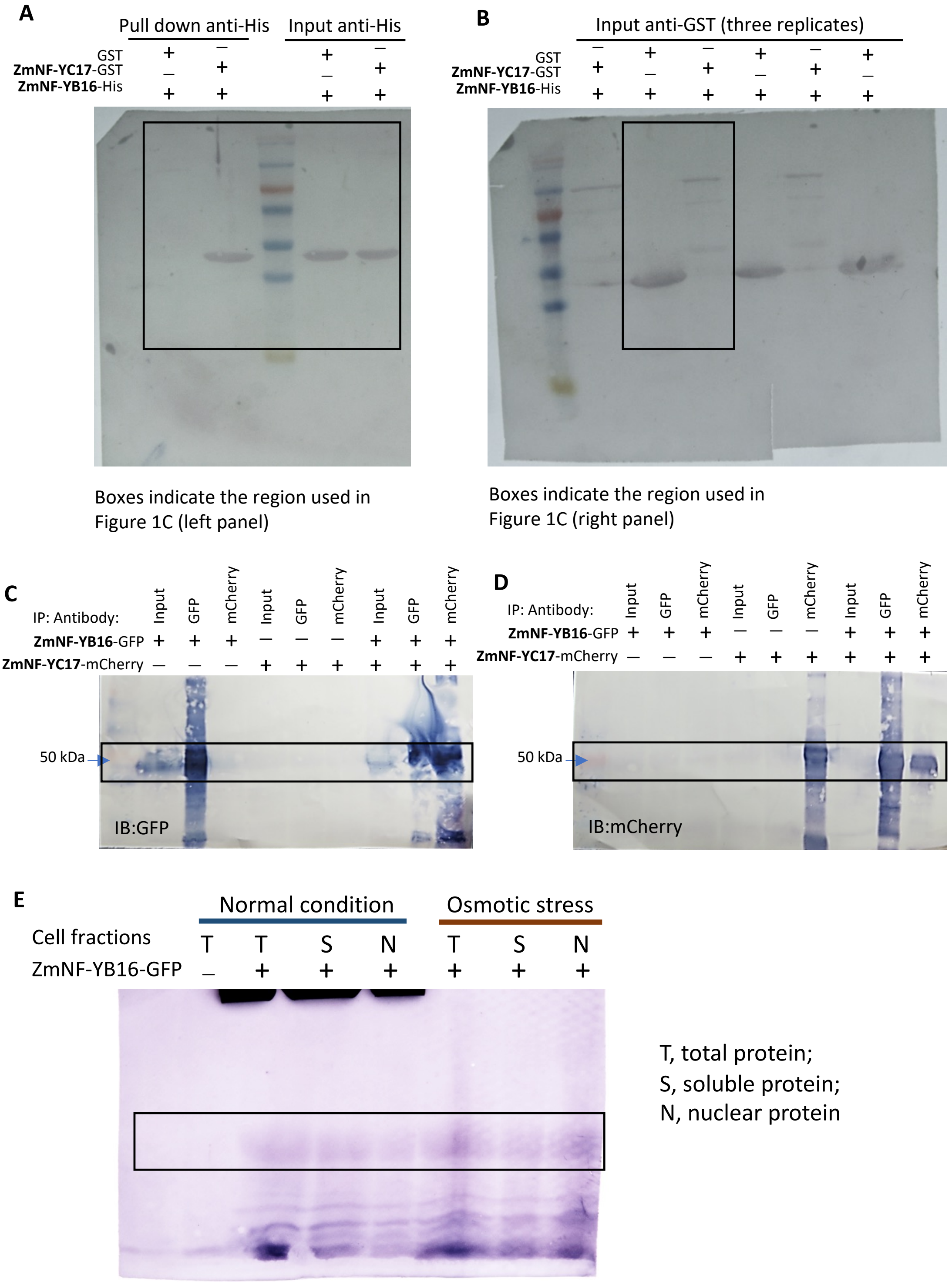

Supplemental Figure S1 The original images of pull-down, Co-IP and western-blot analyses used in this paper

The original images of pull-down in **Fig. 1C** (**A** and **B**), Co-IP in **Fig. 1E** (**C** and **D**) and western-blot in **Fig. 2E** (**E**) used in this paper. Boxes in each panel indicate the regions used for the corresponding figures.

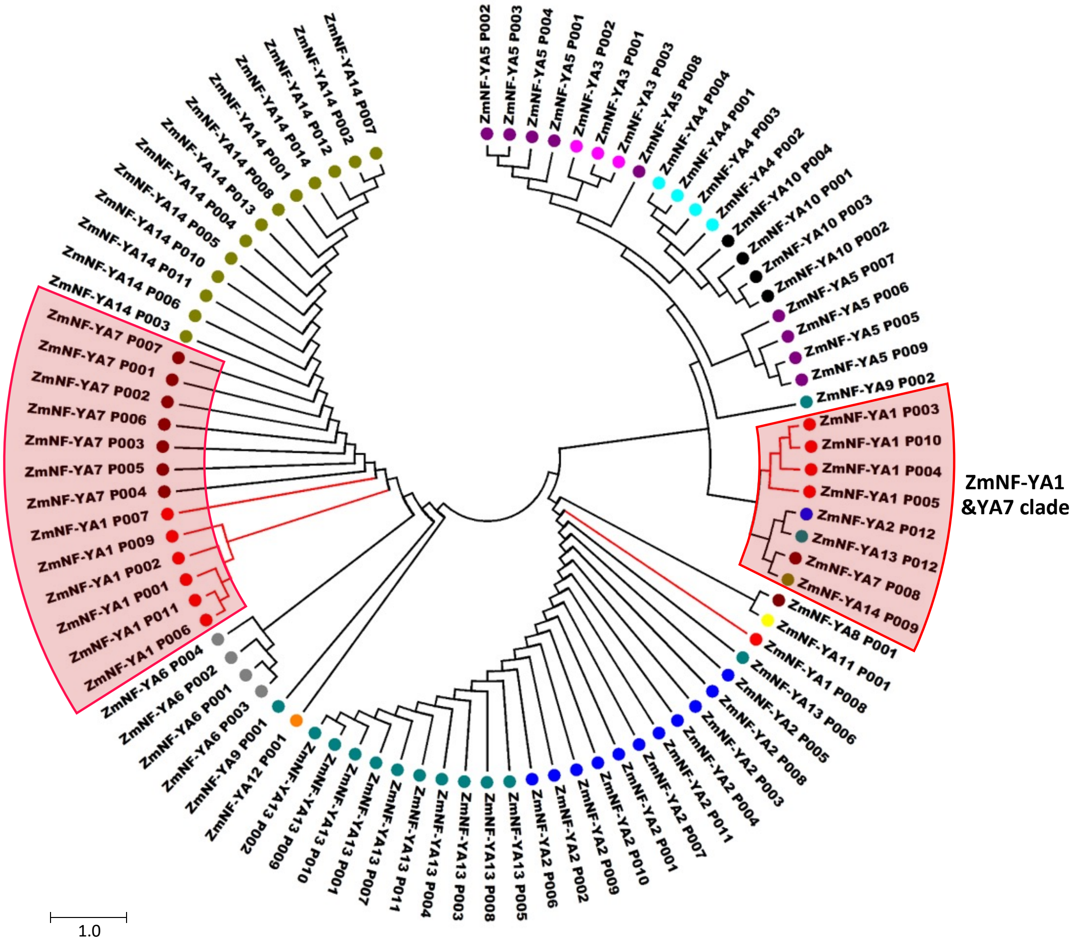

Supplemental Figure S2 Phylogenetic analysis of NF-YA family members of maize

All the transcripts were obtained from MaizeGDB database.
ZmNF-YA1, GRMZM2G000686; ZmNF-YA2, GRMZM2G361842;
ZmNF-YA3, GRMZM5G853836; ZmNF-YA4, GRMZM5G829103;
ZmNF-YA5, GRMZM5G865626; ZmNF-YA6, GRMZM2G091964;
ZmNF-YA7, GRMZM2G040349; ZmNF-YA8, GRMZM2G096016;
ZmNF-YA9, GRMZM2G104396; ZmNF-YA10, GRMZM2G165488;
ZmNF-YA11, GRMZM2G582893; ZmNF-YA12, GRMZM5G857944;
ZmNF-YA13, GRMZM2G038303; ZmNF-YA14, GRMZM2G126957.

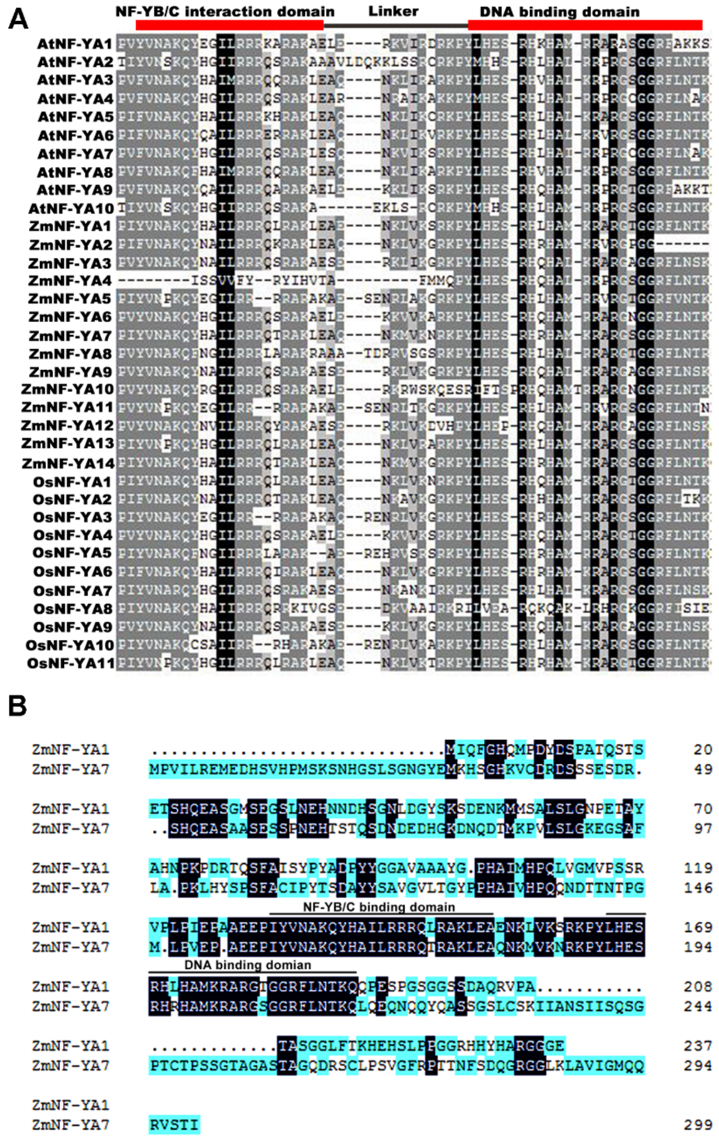

Supplemental Figure S3 Multiple sequence alignment of NF-Y family members from maize and rice

(A) Alignment of amino acid sequences of NF-YA family members and predicated conserved domains in NF-YA structure. The consensus "NF-YB/C interaction domain" and "DNA binding domain" are boxed in red. Conserved amino acid residues are colored in black or gray according to the degree of conservation. **(B)** Sequence alignment of ZmNF-YA1 and ZmNF-YA7.

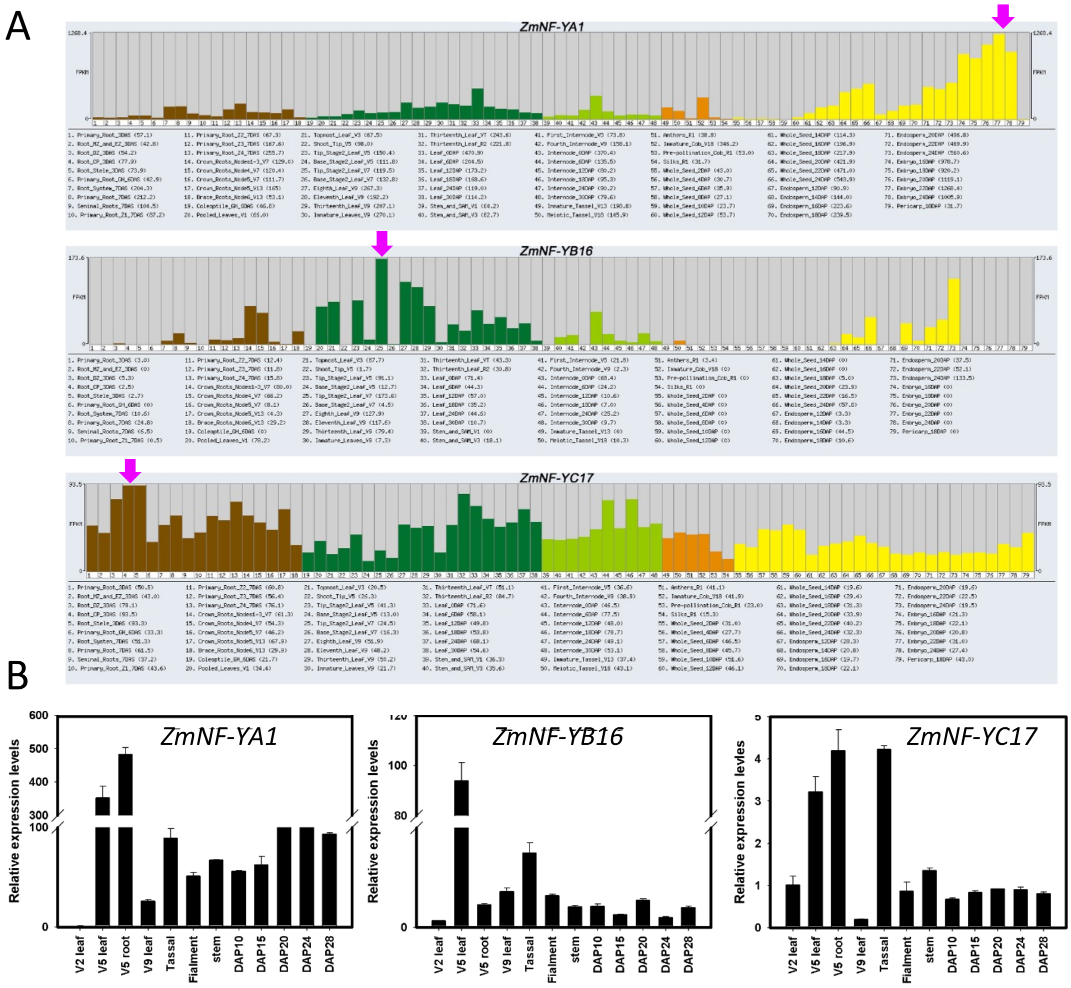

Supplemental Figure S4 Organ expression patterns of the genes related to the heterotrimer

(A) Expression of the *ZmNF-YA1*, *ZmNF-YB16* and *ZmNF-YC17* in 79 distinct replicated samples from maize inbred line B73. Expression data were downloaded from MaizeGDB database (https://www.maizegdb.org) according to the published works (Winter et al., 2007; Sekhon et al., 2011; Stelpflug et al., 2016). **(B)** Real-Time quantitative Reverse Transcription (qPCR) analysis of *ZmNF-YA1*, *ZmNF-YB16* and *ZmNF-YC17* in different tissues and development stages. Relative gene expression levels were calculated with the $2^{-\Delta\Delta Ct}$ method using maize Actin1 as an internal control. The value of expression level in V2 leaf was considered as 1-fold. Error bars represent SD of three independent experiments.

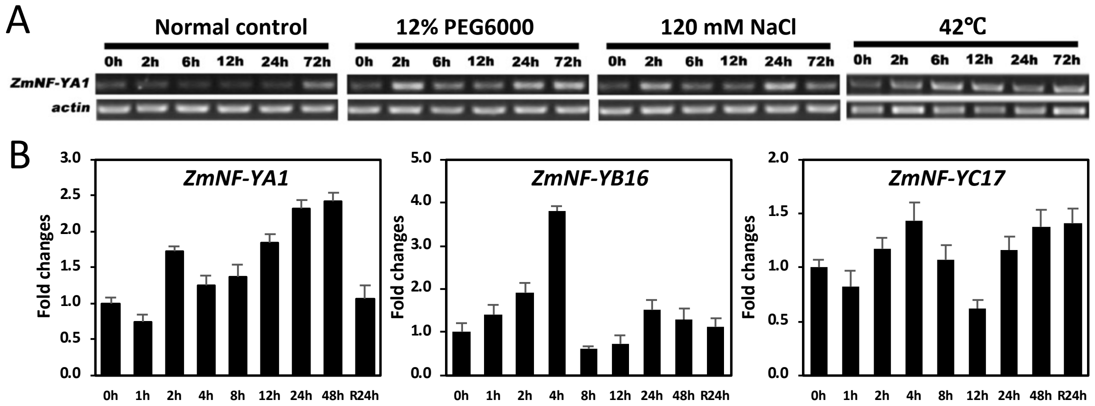

Supplemental Figure S5 Expression profiles of the NF-Y complex members under different abiotic stresses

(A) Semi-quantitative RT-PCR analysis of ZmNF-YA1 in response to osmotic stress (12% PEG6000), 120 mM NaCl and 42 °C heat stress and normal conditions. **(B)** qRT-PCR analysis of ZmNF-YA1, ZmNF-YB16 and ZmNF-YC17 under 12% PEG6000 treatment. Maize seedlings at 3-leaf stage were used for gene expression analysis under different stress treatment and recover for 24 h (R24h). Leaves were collected after stress treatment as indicated. Relative gene expression levels were calculated with the $2^{-\Delta\Delta Ct}$ method using maize Actin1 as an internal control. The value of expression level before stress treatment (0) was considered as 1-fold. Error bars represent SD of three independent experiments.

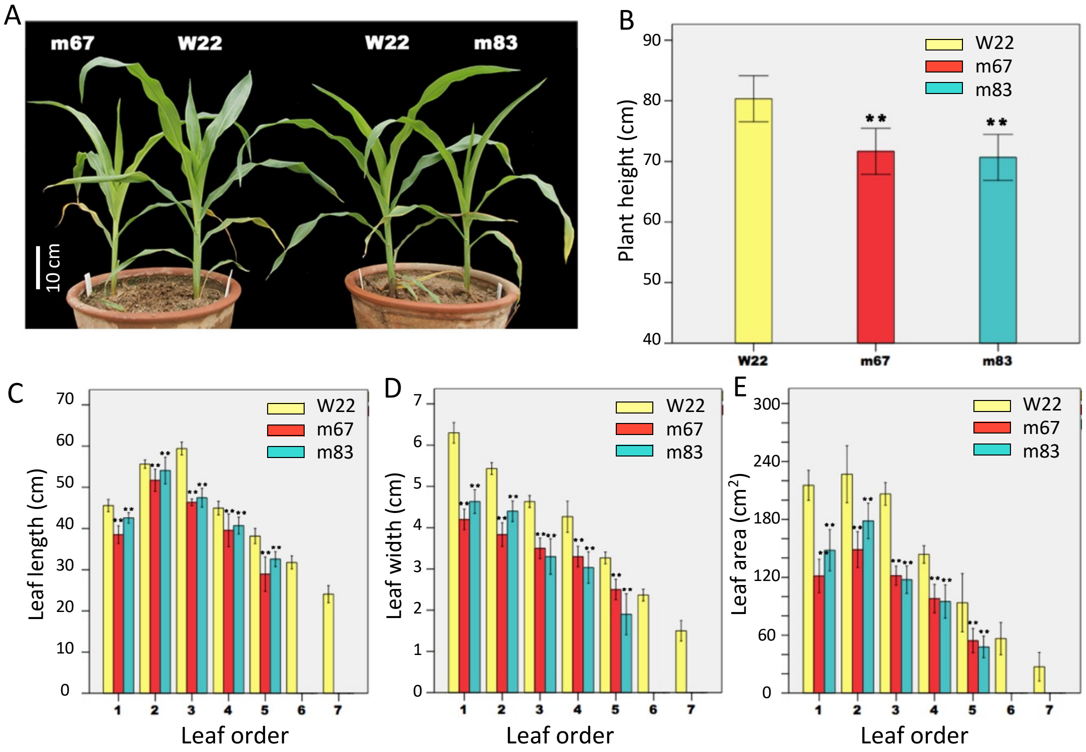

Supplemental Figure S6 *ZmNF-YA1* regulates plant growth in maize.

(A) Phenotypes of W22 and *zmnf-ya1* mutant plants grown under normal conditions. **(B)-(E)** Plant height, leaf length, leaf width and leaf area of W22 and *zmnf-ya1* mutant plants grown under normal conditions. Values are means ± SD (n=5). Statistically differences are indicated by ** (P < 0.01) used Student's *t*-test. m67 (uniformMU number: UFMu-06468) and m83 (uniformMU number: UFMu-02216) were the two Mu insertion lines of *ZmNF-YA1*.

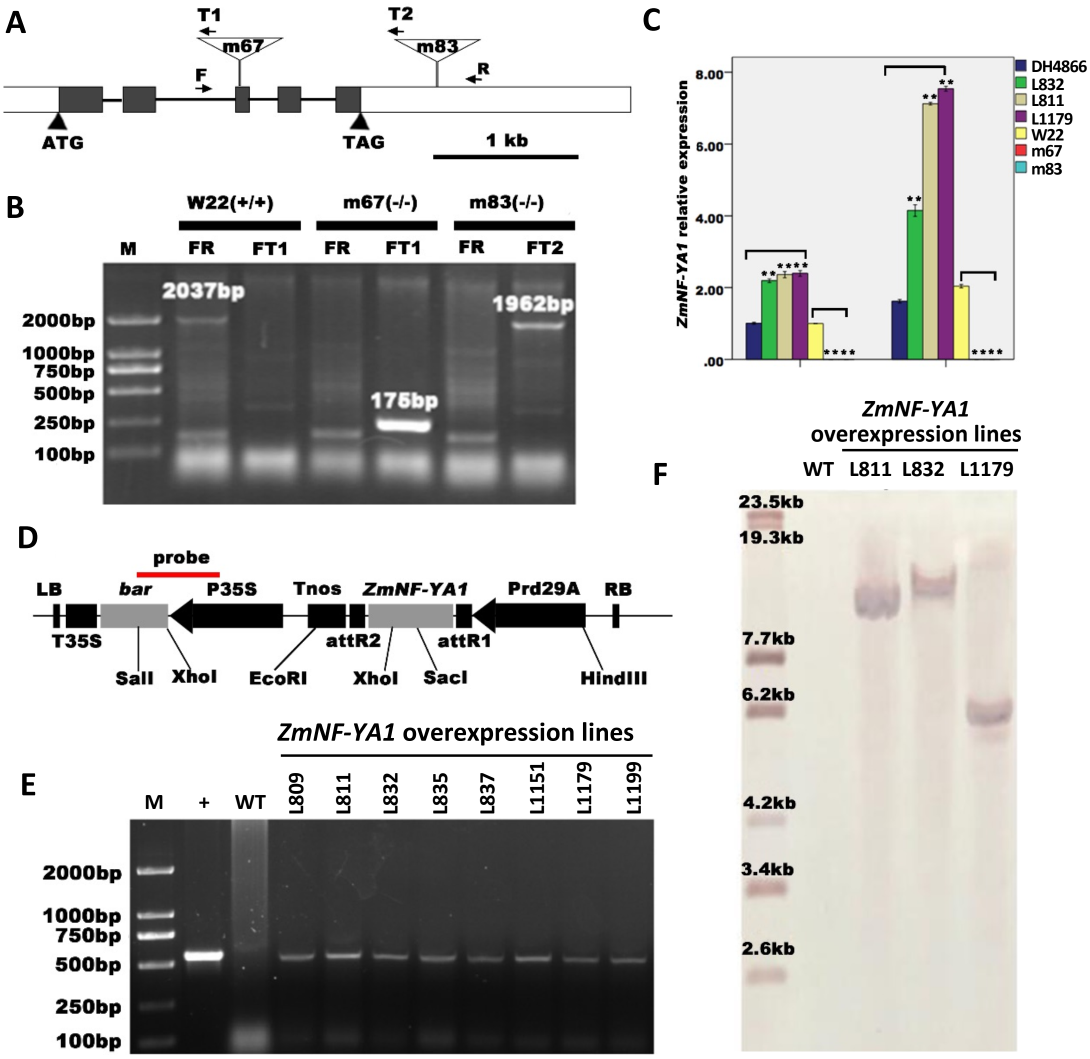

Supplemental Figure S7 Molecular characterization of *zmnf-ya1* mutants and *ZmNF-YA1* overexpression transgenic plants

(A) The *ZmNF-YA1* gene structure and the Mu insertion sites in two alleles named as m67 (uniformMU number: UFMu-06468) and m83 (uniformMU number: UFMu-02216). Exons are designated as boxes and intron as solid lines. The translation regions are marked with filled boxes. PCR primer anchor sites are indicated as arrows. **(B)** PCR analysis of *zmnf-ya1* mutants. F and R are primers specific to *ZmNF-YA1*. T1 and T2 primers are specific to m67 and m83. +/+ and -/- designated WT (W22) and homozygote for the *zmnf-ya1* mutants, respectively. **(C)** *ZmNF-YA1* expression levels in the leaves of *ZmNF-YA1* OE, *zmnf-ya1* and their correspondent wild-type DH4866 and W22 plants without (left) or with 12% PEG6000 treated for 12 h (right). The value of *ZmNF-YA1* expression level in WT was considered as 1-fold. Error bars represent SD of three independent experiments and asterisks indicate a significant difference (** P<0.01) compare with the corresponding WT plants. **(D)** The T-DNA region of the transformed construct pB7WG2.0-Prd29A::*ZmNF-YA1*-P35S::*bar*. The vector includes a *ZmNF-YA1* gene driven by Prd29A, an abiotic stress-responsive promoter from

Arabidopsis, and a *bar* gene driven by P35S, a promoter of CaMV35S from cauliflower mosaic virus. The specific probe for southern blotting analysis is boxed in red. **(E)** PCR analysis of the *ZmNF-YA1* overexpressing T3 transgenic maize for the *bar* gene. M, DNA marker DL2000; +, the positive control using the plasmid pB7WG2.0-Prd29A::*ZmNF-YA1*-P35S::*bar* as a template; WT, untransformed control DH4866; L809, L811, L832, L835, L837, L1151, L1179 and L1199, different *ZmNF-YA1* transgenic T3 lines. **(F)** Southern blotting analysis of the *ZmNF-YA1* transgenic T3 plants. The genomic DNA was digested with *Eco*R I. A DIG-labelled fragment spanning P35S and *bar* was used as specific probe. M, **λ**-DNA/*EcoT*14 molecular weight marker; WT, wild-type DH4866; L811, L832 and L1179, T3 transgenic plants overexpressing *ZmNF-YA1*.

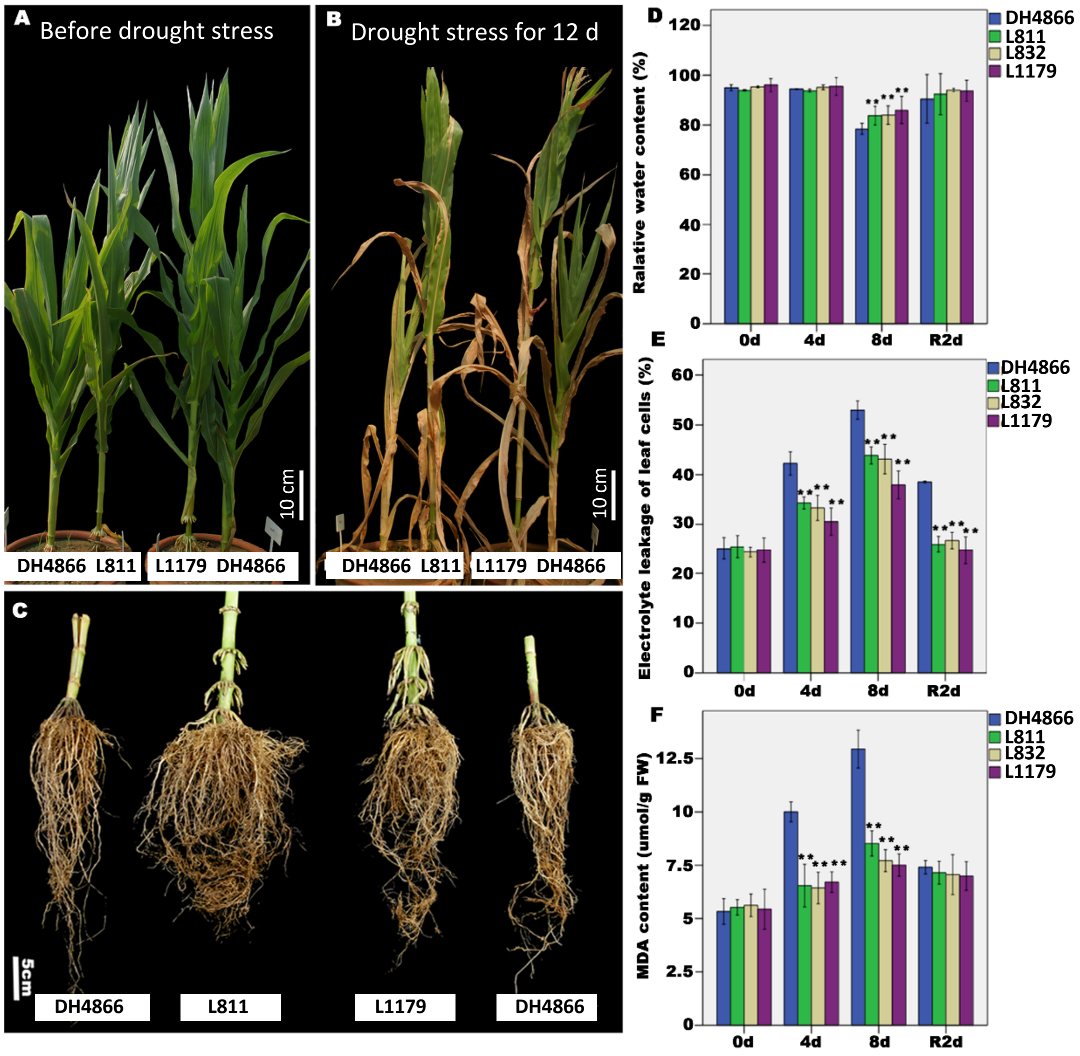

Supplemental Figure S8 Overexpression of *ZmNF-YA1* confers the drought tolerance of maize plants in the fields

(A) and **(B)** Representative phenotypes of DH4866 and *ZmNF-YA1* OE plants grown in pots at pre-flowering stage before and after 12 days (d) of drought stress. The images of panel **A** were digitally extracted for comparison. **(C)** Root systems of DH4866 and *ZmNF-YA1* OE plants after 12 d of drought stress. **(D)-(F)** Relative water content, electrolyte leakage and malondialdehyde (MDA) content of leaves from plants with different duration of water-withholding. L811, L832 and L1179 were the *ZmNF-YA1* overexpression lines. Maize inbred line DH4866 was the wild type. Values are means of three biological replicates ± SD. Statistically significant differences determined by Student's *t*-test: **, $P < 0.01$.

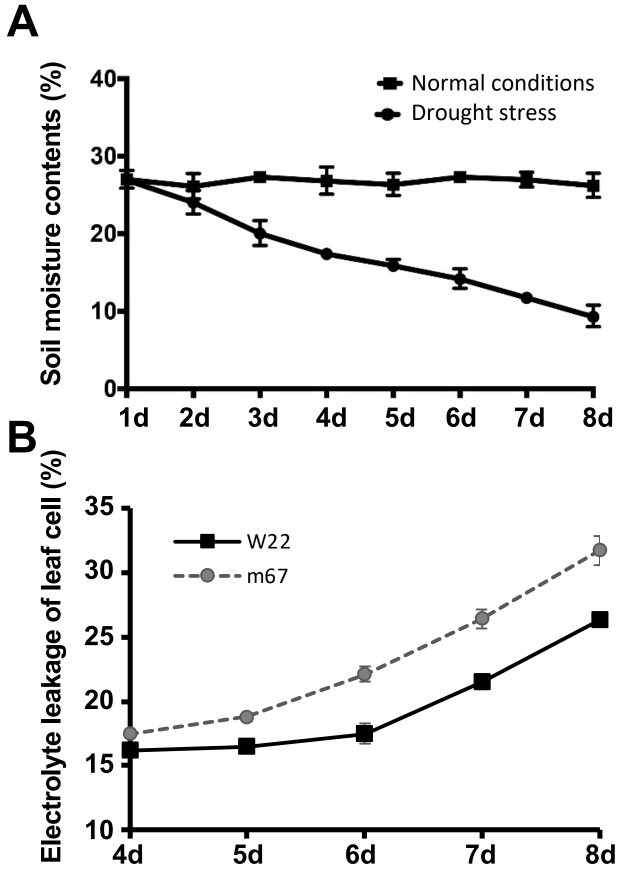

Supplemental Figure S9 Measurement of physiological index of samples for RNA-sequencing

(A) Soil moisture contents were determined since the 1st day of water withholding. The two conditions were the normal conditions and drought stress respectively. **(B)** The electrolyte leakage of leaf cell from plants subjected to drought stress from the 4 days (d) to 8 d. Values are means of three biological replicates ± SD (n=15). Statistically significant differences determined by Student's t-test: **, P < 0.01. m67: *zmnf-ya1* mutant line. W22 were the wild type control.

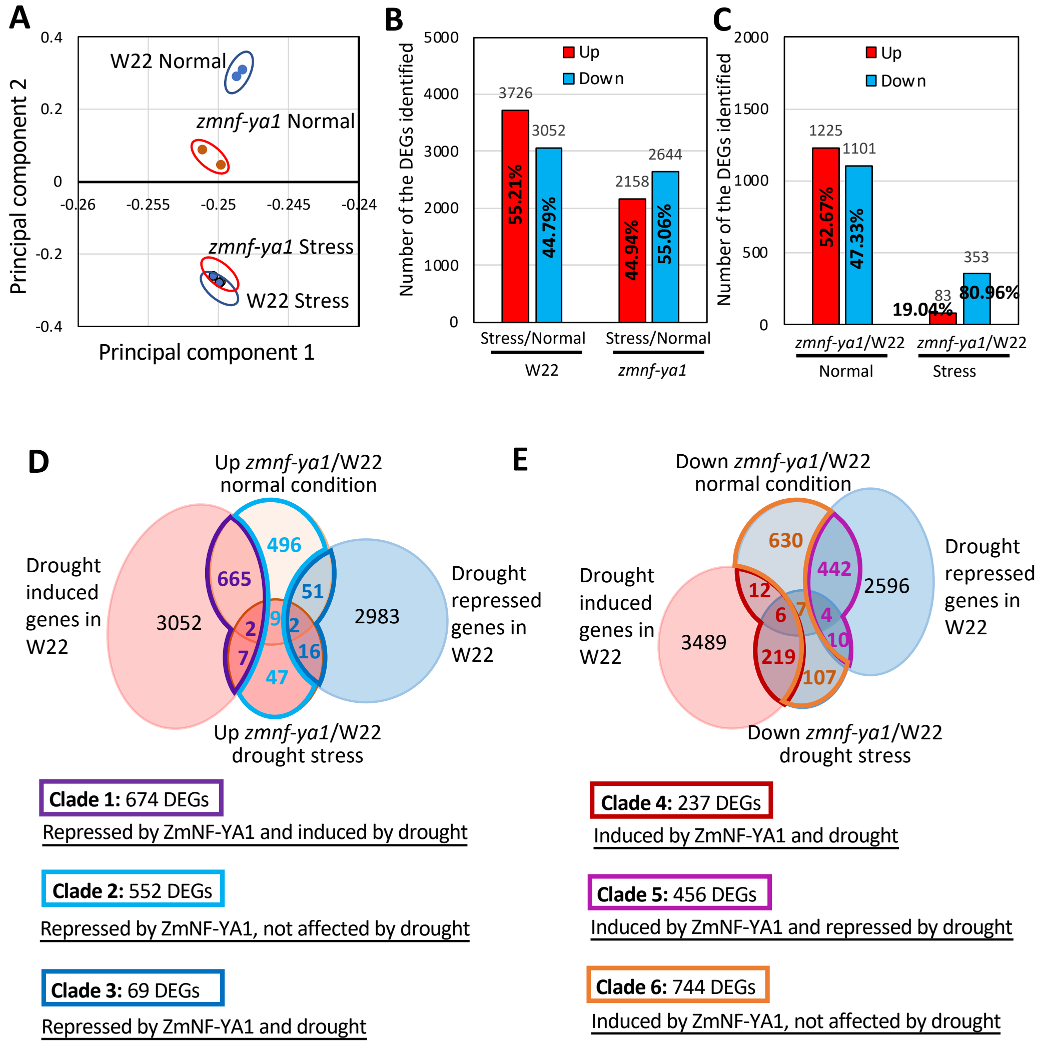

Supplemental Figure S10 Overview of the RNAseq analysis and the six different clades DEGs regulated by ZmNF-YA1

(A) Principal component analysis (PCA) of data from the RNAseq analysis. **(B)** and **(C)** Differentially expressed genes (DEGs) in the comparison between different lines and treatment. Normal in the plot indicates normal control and stress indicates drought stress treatment used. P value (adjusted p-value) < 0.05 and absolute log2 ratio > 1 were used as cutoffs for significant differential expression. **(D)** and **(E)** Venn diagram of the DEGs in different genotypes under normal and drought stress. Based on their expression patterns, the DEGs affected by the interaction of ZmNF-YA1 and drought stress and the ZmNF-YA1 alone can be grouped into six different clades. The upregulated DEGs in the *zmnf-ya1* mutant (ie, repressed by ZmNF-YA1, panel **(D)** were grouped into Clades 1-3 (674 DEGs, 552 DEGs and 69 DEGs) based on if how they response to drought stress **(D)** The upregulated DEGs in the *zmnf-ya1* mutant (ie, induced by ZmNF-YA1, panel **(E)** were grouped into Clades 4-6 (237 DEGs, 456 DEGs and 744 DEGs) based on if how they response to drought stress **(E)** The colors in same clade were boxed with the same color as that in the vein diagram.

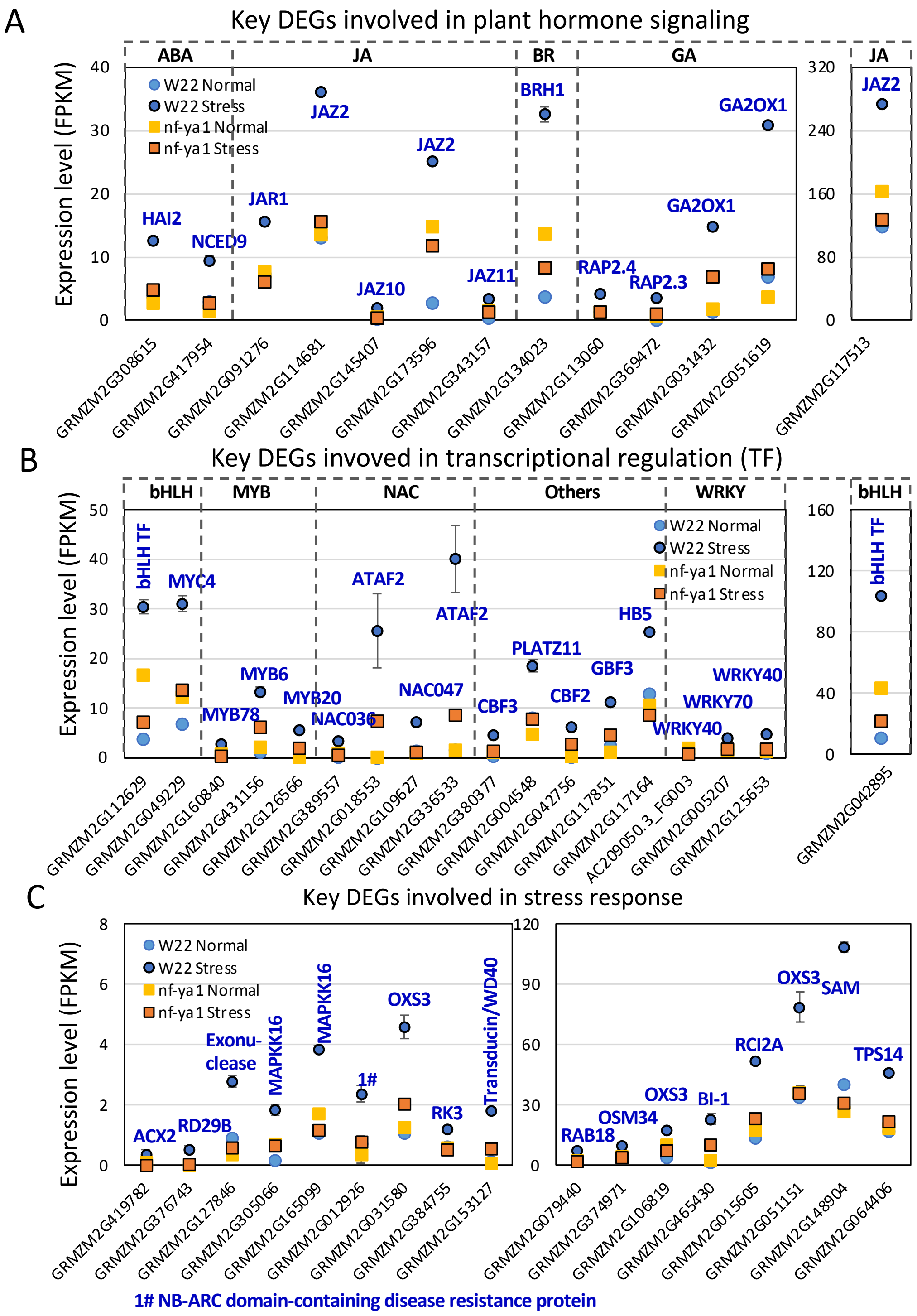

Supplemental Figure S11 Expression of the key DEGs in the comparisons of the *zmnf-ya1* mutant versus W22

Expression of the key DEGs involved in plant hormone signaling **(A)**, transcriptional regulation **(B)** and stress response **(C)** in the *zmnf-ya1* (*nf-ya1*) mutant and W22 under normal control and drought stress conditions. Dot plots show the relative expression levels (Fragments Per Kilobase of transcript per Million mapped reads) of the key genes.

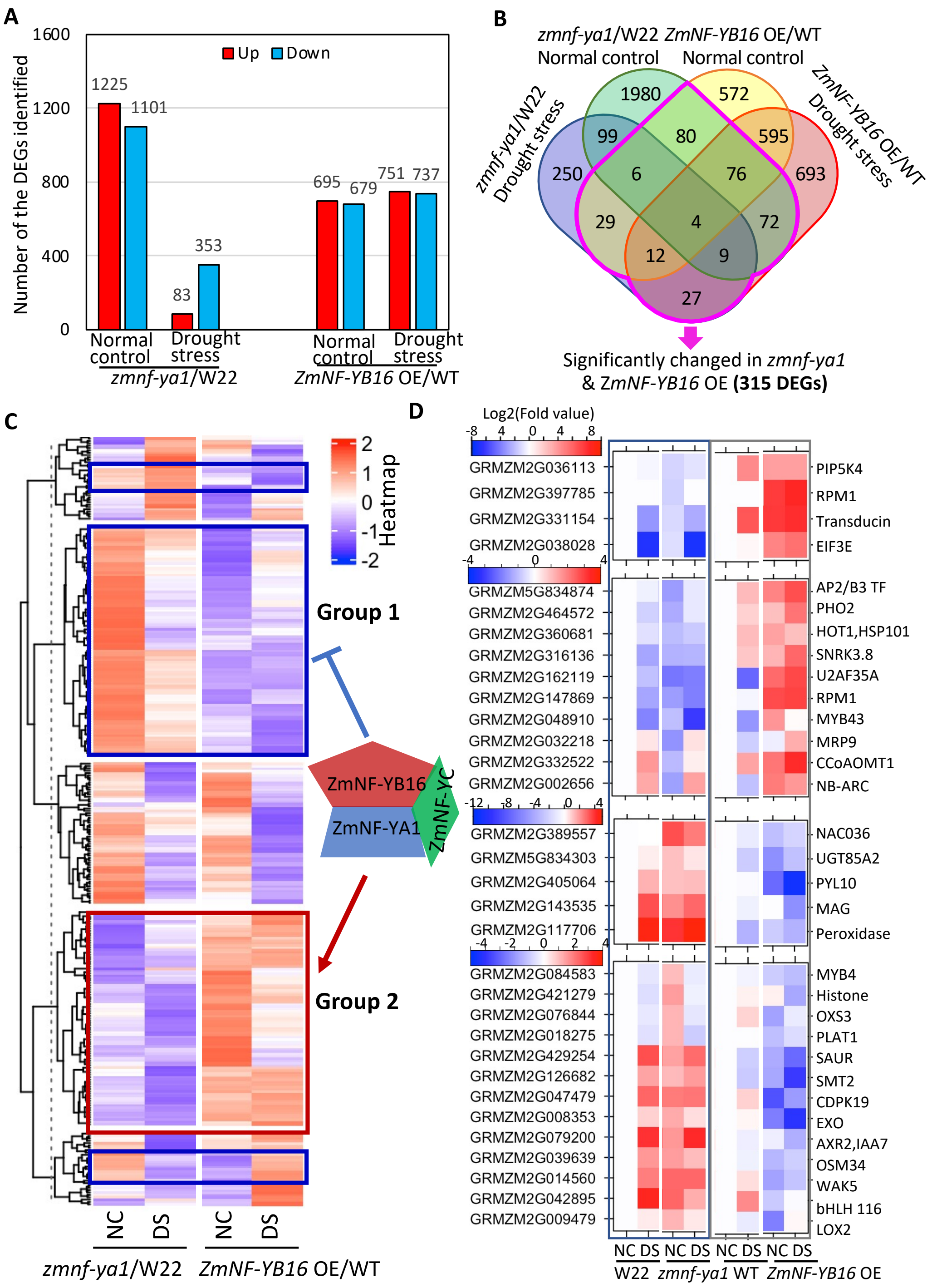

Supplemental Figure S12 Comparative transcriptome analysis of ZmNF-YA1 and ZmNF-YB16-regulated genes

(A) Numbers of genes with differential expression between the *zmnf-ya1* mutant and W22, the *ZmNF-YB16* overexpression lines (OE) and its wild type control B104 under normal and drought stress conditions. (B) Venn diagram of the DEGs in *ZmNF-YA1* and *ZmNF-YB16*-regulated genes. From the vein plot, 315 DEGs affected by *ZmNF-YA1* and *ZmNF-YB16* were identified. (C) K-mean cluster analysis of the 315 DEGs identified in panel (B). Based on their expression patterns, those DEGs can be grouped

into different groups. Group 1 (blue line boxed) and Group 2 (auburn line boxed) were the major types of the DEGs regulation by ZmNF-YA1 and ZmNF-YB16. The expression levels of Group 1 DEGs were higher in the *zmnf-ya1* mutant than W22 and lower in the *ZmNF-YB16* OE compared with B104, while opposite trends for Group 2 DEGs. NC, normal control and DS, drought stress. (D) Heatmap of the representative DEGs in Group 1 and Group 2 DEGs in the *zmnf-ya1* mutant and W22, the *ZmNF-YB16* OE and its B104 under normal and drought stress conditions. Log2 fold value were used to generate the heatmap, and all of them compared with the value in wild type. NC, normal control and DS, drought stress.

Supplemental materials and methods

Identification of *zmnf-ya1*mutants

Two *zmnf-ya1* mutants were isolated from the UniformMu population in the W22 background (McCarty et al., 2005). PCR was used to identify the mutants using the Mu-TIR primer and *ZmNF-YA1* specific primer **(Table S8).** T3 (self-pollinated for 3 generations) mutants were used for this study.

Transformation of maize and production of transgenic plant lines

Elite maize inbred line DH4866 shoot tips were transformed with *Agrobacterium tumefaciens* strain LBA4404 (Li et al., 2008) containing a mini-Ti pB7WG2.0-Prd29A::*ZmNF-YA1*-P35S::*bar*. The T-DNA region contains the coding sequence of the *ZmNF-YA1* gene driven by an *Atrd29A* promotor from Arabidopsis (Yamaguchi-Shinozaki and Shinozaki, 1994) and a phosphinothricin acetyltransferase gene (*bar*) that confers resistance to the herbicide glufosinate ammonium (De Block et al., 1987). The transformed plantlets were screened at the 3-leaf stage by spraying them with Liberty® 280 SL herbicide [containing 24.5% (v/v) glufosinate-ammonium, Willowood, USA] at a 0.25% (v/v) final concentration, and the surviving seedlings were chosen for PCR analysis. The herbicide-resistant and PCR-positive seedlings were transplanted in the field and self-pollinated to produce the next generation. The transgenic plants were screened with herbicide and verified by PCR for generations.

PCR assay and southern blot analysis

Genomic DNA was extracted from young maize leaves according to the cetyltrimethylammonium bromide (CTAB) method. The leaves were sampled from the

PCR-positive *ZmNF-YA1* overexpression T3 plants and WT DH4866. The DNA was digested with *Eco*R I for southern blotting with a digoxigenin (DIG)-labeled specific probe according to the DIG system manual (1636090, Roche, Shanghai, China). The probe was a fragment of pB7WG2.0-Prd29A::*ZmNF-YA1*-P35S::*bar* spanning P35S and the *bar* gene.

RNA extraction and RT-qPCR

Total RNA was extracted from plant samples using TRIzol reagent (Sangon Biotech, Shanghai, Chian) and cDNA synthesis with the RT reagent kit was performed according to the manufacturer's protocol (DRR420, TaKaRa, Dalian, China). Expression patterns were detected by RT-qPCR. RT-qPCR was conducted with the SYBR® RT-PCR Kit (RR037Q, TaKaRa, Dalian, China) using 25 μl reactions for each sample and amplified through 40 cycles on a LightCycler 480 (Roche, Basel, Switzerland). *ZmActin* was chosen as an internal control. The primers used for RT-qPCR are listed in Table S8. The relative gene expression levels were calculated by the $2^{-\Delta\Delta Ct}$ method (Livak and Schmittgen, 2001). Each experiment was repeated 3 times, and three biological replicates was used.

Stress treatment of maize plants

The seeds of different lines were surface sterilized with 70% (v/v) ethanol and 0.1% (v/v) $HgCl_2$ solution and washed with sterilized water. The sterilized seeds were germinated on moist filter paper at 28℃ for 3 days (d) and then transferred into hydroponic culture boxes containing 30 L of nutrient solution (Li et al., 2011). The nutrient solution was replaced every 3 d to maintain a pH of 6.0±0.1. The seedlings were grown at 32/25℃ with a 14-h-light/10-h-dark cycle in a greenhouse. Uniform seedlings at the V3 stage (11 d old) were used for stress treatments with or without 12% (v/v) PEG_{6000}, 120 mM NaCl or 15 mM LiCl in nutrient solution. After 10 days of dehydration stress, salt stress or ion toxic treatments, the plants were harvested to measure the biomasses and morphological parameters. Three independent replicates were assessed, and 10-15 individual plants in each replicate.

The drought stress treatment during the pre-flowering stage was performed by pot cultivation under a rainproof shelter in Jinan, China. Three independent *ZmNF-YA1* T3 transgenic lines and non-transgenic DH4866 were sown in pots (diameter: 35 cm; height: 30 cm) with equal amounts of soil in May. At the V3 stage, the plants were

thinned to one seedling per pot. At the V12 stage, the plants were divided into two groups for drought stress treatment by controlling the watering to maintain the soil moisture content at approximately 10%. One group was subjected to continuous drought stress treatment for 12 d for phenotype analysis; the other group was treated for 8 d and then exposed to rehydration treatment by sufficient irrigation. The samples were collected from ear leaf, and then the physiological parameters were measured, including the relative water content (RWC), electrolyte leakage and malondialdehyde (MDA) concentration, at 0, 4 and 8 d during drought stress treatment and at 2 d of rehydration treatment. Measurement of RWC, electrolyte leakage, MDA content and total soluble sugar was performed same as that Quan et al (Quan et al., 2004) and Li et al (Li et al., 2008). The anthrone method was used to estimate the carbohydrates in different samples (Yemm and Willis, 1954).

RNA-sequencing and differential gene expression analysis

Wild-type (W22) and *zmnf-ya1* (m67) were grown in pots in a greenhouse. Maize plants were grown under well-watered conditions until the V4 stage, and then half of them were exposed to drought stress treatment. Water loss in the soil was determined using a soil moisture content analyzer (TOP instrument, Zhejiang, China). Electrolyte leakage from leaf cells was used to assess the degree of drought stress in plants. The time of sampling was determined by monitoring the soil moisture content and leaf cell electrolyte leakage. The samples were named WC (W22-Control), WS (W22-stress), MC (*zmnf-ya1*-control) and MS (*zmnf-ya1*-stress), and the top three leaves were collected and immediately frozen in liquid nitrogen. Leaves from 3-4 plants were pooled for each sample, and two replicates were used in the study.

For comparing the ZmNF-YB16 OE line vs B104, data and treatment are from our previously published papers (Wang et al., 2018; Wang et al., 2019). Drought stress treatment of plants was performed in the maize growing season as described in our previous report (Wang et al., 2019). The trial plots (4 rows/plot, 10 plants/row, 66, 700 plants/ha) were arranged in a randomized complete block design with four replications. A 30 cm impermeable zone between plots (30-cm-deep embedded plastic sheeting) was set up. All the plants were grown under natural conditions (200–1600 mmolm^{-2} s^{-1} at noontime, 20–35 °C, normal nutrients, well-watered with soil water content ≥19.5%) until the designated stages. Soil water content was monitored by using a Soil Moisture Content Meter (TZS, TOP instrument, China). For drought stress at the 5 DAP stage,

plants were grown under natural conditions until the pollination stage, and then they were subjected to drought stress treatment by maintaining 14.0–15.0% SWC, followed by the restoration of watering. Ear leaf from the plants at 5 DAP that had suffered 5 days of drought treatment and the control materials were sampled for RNA sequencing. Leaves from 3-4 plants were pooled for each sample, and two replicates were used.

Total RNA was isolated using TRIzol (Invitrogen, USA for *zmnf-ya1* vs W22) or McCarty (McCarty, 1986) for the *ZmNF-YB16* vs B104 because of the high sugar content in tissue. The cDNA libraries were constructed according to BGI standard protocols and sequenced using BGISEQ500 (BGI, Shenzhen, China) for *zmnf-ya1* mutant and W22 and Illumina HiSeq200 for *ZmNF-YB16* OE lines and B104. RNA-sequencing reads were mapped to the maize genome (V3 5b+) with HISAT (version 2.0.4) (Kim et al., 2015) after filtering out low-quality reads using SOAPnuke (version 1.5.2) (Chen et al., 2018). Clean reads were mapped to the reference database using Bowtie2 (version 2.2.5) (Langmead and Salzberg, 2012), and then gene expression levels were calculated with RSEM (version 1.2.12) (Li and Dewey, 2011). The FPKM value (fragments per kb per million reads) was used to normalize the gene expression level. Differentially expressed genes (DEGs) between each pair of samples were detected with DEseq2 algorithms (Love et al., 2014). A resulting P value (adjusted p-value) < 0.05 and fold change value > 2 (log2 ratio > 1) were used as cutoffs for significant differential expression (GEO Submission (GSE137780)). Gene functional annotations were conducted based on MaizeGDB (284 Zea mays release of Phytozome 10) (https://www.maizegdb.org). GO enrichment analysis was based on the AGRIGO database (http://bioinfo.cau.edu.cn/agriGO/) and the GENEONTOLOGY (http://geneontology.org).

Literature Cited in this Supplemental material and methods

De Block M, Botterman J, Vandewiele M, Dockx J, Thoen C, Gosselé V, Movva NR, Thompson C, Van Montagu M, Leemans J (1987) Engineering herbicide resistance in plants by expression of a detoxifying enzyme. EMBO J **6**: 2513–2518

Chen Y, Chen Y, Shi C, Huang Z, Zhang Y, Li S, Li Y, Ye J, Yu C, Li Z, et al (2018) SOAPnuke: A MapReduce acceleration-supported software for integrated quality control and preprocessing of high-throughput sequencing data. Gigascience **7**: 1–

6

Kim D, Langmead B, Salzberg1 SL (2015) HISAT: a fast spliced aligner with low memory requirements Daehwan HHS Public Access. Nat Methods **12**: 357–360

Langmead B, Salzberg SL (2012) Fast gapped-read alignment with Bowtie 2. Nat Methods **9**: 357–359

Li B, Dewey CN (2011) RSEM: Accurate transcript quantification from RNA-Seq data with or without a reference genome. BMC Bioinformatics. doi: 10.1186/1471-2105-12-323

Li B, Wei A, Song C, Li N, Zhang J (2008) Heterologous expression of the TsVP gene improves the drought resistance of maize. Plant Biotechnol J **6**: 146–159

Li Z, Gao Q, Liu Y, He C, Zhang X, Zhang J (2011) Overexpression of transcription factor ZmPTF1 improves low phosphate tolerance of maize by regulating carbon metabolism and root growth. Planta **233**: 1129–1143

Livak KJ, Schmittgen TD (2001) Analysis of relative gene expression data using real-time quantitative PCR and the 2-ΔΔCT method. Methods **25**: 402–408

Love MI, Huber W, Anders S (2014) Moderated estimation of fold change and dispersion for RNA-seq data with DESeq2. Genome Biol **15**: 1–21

McCarty DR (1986) A simple method for extraction of RNA from maize tissues. Maize Genet Coop Newslett **60:** 61

McCarty DR, Mark Settles A, Suzuki M, Tan BC, Latshaw S, Porch T, Robin K, Baier J, Avigne W, Lai J, et al (2005) Steady-state transposon mutagenesis in inbred maize. Plant J **44**: 52–61

Quan R, Shang M, Zhang H, Zhao Y, Zhang J (2004) Engineering of enhanced glycine betaine synthesis improves drought tolerance in maize. Plant Biotechnol J **2**: 477–486

Wang B, Li Z, Ran Q, Li P, Peng Z, Zhang J (2018) ZmNF-YB16 overexpression

improves drought resistance and yield by enhancing photosynthesis and the antioxidant capacity of maize plants. Front Plant Sci **9**: 709

Wang B, Liu C, Zhang D, He C, Zhang J, Li Z (2019) Effects of maize organ-specific drought stress response on yields from transcriptome analysis. BMC Plant Biol **19**: 1–19

Yamaguchi-Shinozaki K, Shinozaki K (1994) A novel cis-acting element in an arabidopsis gene is involved in responsiveness to drought, low-temperature, or high-salt stress. Plant Cell **6**: 251–264

Yemm BEW, Willis AJ (1954) The Estimation of Carbohydrates in Plant Extracts by Anthrone. Biochem J **57**: 508–514
